# Global burden of prostate cancer: age-period-cohort analysis from 1990 to 2021 and projections until 2040

**DOI:** 10.1186/s12957-025-03733-1

**Published:** 2025-03-20

**Authors:** Feifan Chu, Lumin Chen, Qing Guan, Zujie Chen, Qiwei Ji, Yuning Ma, Jinzhong Ji, Mingxin Sun, Tingyang Huang, Haihan Song, Hao Zhou, Xiuquan Lin, Yichun Zheng

**Affiliations:** 1 Department of Urology, International School of Medicine, The Fourth Affiliated Hospital of School of Medicine, International Institutes of Medicine, Zhejiang University, Yiwu, 322000 China; 2 School of Health Management, Fujian Medical University, Fuzhou, Fujian 350122 China; 3 School of Economics and Management, Beijing University of Technology, Beijing, 100124 China; 4 Central Lab, Shanghai Key Laboratory of Pathogenic Fungi Medical Testing, Shanghai Pudong New Area People’s Hospital, Shanghai, China; 5 Department of Immunology, DICAT National Biomedical Computation Centre, Vancouver, BC Canada; 6 Department for Chronic and Noncommunicable Disease Control and Prevention, Fujian Provincial Center for Disease Control and Prevention, Fuzhou, 350012 Fujian China

## Abstract

**Background:**

Prostate cancer (PCa) is the second most common cancer among men worldwide. This study uses data from the 2021 Global Burden of Disease (GBD) study to estimate the global burden of prostate cancer from 1990 to 2021.

**Methods:**

We analyzed the incidence, mortality, and disability-adjusted life years (DALYs) of prostate cancer globally from 1990 to 2021. Based on the Sociodemographic Index (SDI), we used the estimated annual percentage change (EAPC) and Age-Period-Cohort model to compare the burden of disease across different age groups and regions with varying levels of development. Finally, we used the Bayesian Age-Period-Cohort model to predict the trend of changes in the disease burden of prostate cancer by 2040.

**Results:**

In 2021, the global age-standardized incidence rate (ASIR) of prostate cancer was 15.37 per 100,000, an increase from 13.69 per 100,000 in 1990. However, the age-standardized mortality rate (5.26 per 100,000) and DALY rate (95.94 per 100,000) decreased significantly compared to 1990. The burden of prostate cancer increased with age, but overall, the burden across all age groups was lower in 2021 than in 1990. The only exception was the incidence rate among individuals under 75 in 2021. High-income regions such as North America and Australia exhibited the highest burden in terms of ASIR, though there has been some reduction in recent years. Conversely, the burden of mortality and DALYs was highest in regions such as sub-Saharan Africa, West Africa, and the Caribbean, where rates have continued to rise. Correlation analysis between SDI and the EAPC of the disease burden showed a negative correlation between EAPC of prostate cancer mortality and DALYs with SDI. The APC analysis showed that in 2021, the ASIR of prostate cancer in high SDI regions was still significantly higher across all age groups compared to other regions. In regions with middle SDI and above, the age-standardized mortality rate and DALY rate decreased over time or across birth cohorts, with a faster decline in areas with higher SDI. By 2040, it is projected that the global ASIR of prostate cancer will reverse its current trend and increase, while the age-standardized mortality rate and DALY rate will continue to decline, and the counts of incidence, mortality, and DALYs will keep rising.

**Conclusion:**

Although the global mortality rate and DALY rate for prostate cancer show a decreasing trend, the number of new cases, deaths, and DALYs continues to rise due to global population growth and the aging population, and the disease burden remains significant. Furthermore, there are substantial geographic disparities in the disease burden of prostate cancer. Therefore, targeted programs should be implemented to strengthen prostate cancer diagnosis and treatment in these specific regions.

**Supplementary Information:**

The online version contains supplementary material available at 10.1186/s12957-025-03733-1.

## Introduction

Prostate cancer is a malignant tumor originating from the epithelial tissue of the male prostate gland [1], and it is the most common cancer among men in nearly two-thirds of countries worldwide, as well as the fifth leading cause of cancer-related mortality in men [2]. Prostate cancer typically progresses slowly, with early symptoms often being subtle or absent, leading many patients to be diagnosed at advanced stages [3]. Once prostate cancer reaches an advanced stage and metastasizes, particularly to bones or other organs, the five-year survival rate can be as low as 30.5% [4]. Current treatment options, such as surgery and radiotherapy, often result in complications related to urinary control and erectile function [5]. Additionally, while androgen deprivation therapy (ADT) has emerged as a treatment option, the majority of patients develop resistance after long-term treatment [6, 7]. Taken together, prostate cancer continues to impose a significant disease burden on patients and global health systems.

In addition to its high incidence, the epidemiological characteristics of prostate cancer include its age-related nature and geographical variability. The incidence of prostate cancer is much higher in older populations compared to younger groups [2]. Geographically, according to the latest estimates from the International Agency for Research on Cancer (IARC), developed countries such as the United States and Australia have some of the highest incidence rates globally, while the mortality burden is concentrated in sub-Saharan Africa, West Africa, and the Caribbean [2]. Therefore, it is crucial to formulate public health policies tailored to different populations and regions to alleviate the burden of prostate cancer. However, IARC’s estimates do not account for the burden imposed by disability following the disease, which is why this study uses global data from the Global Burden of Disease (GBD) 2021, focusing on incidence, mortality, and disability-adjusted life years (DALYs) to better describe the disease burden.

The GBD database is derived from the most comprehensive research to date, aimed at quantifying health losses across different periods, regions, and populations to guide progress in health systems. Previous analyses have already been conducted on the global and regional burden of prostate cancer based on the GBD 2019 database [8–10], providing valuable insights for the diagnosis and treatment of prostate cancer. Therefore, this study utilizes the latest 2021 GBD database, combined with the Age-Period-Cohort (APC) model and Bayesian Age-Period-Cohort (BAPC) model, in order to extract and analyze updated, more comprehensive, and deeper epidemiological information on prostate cancer, continuing to contribute to reducing the disease burden.

This study describes the epidemiological trends of prostate cancer across different time periods, geographic locations, and population groups. Furthermore, we investigate the relationship between the estimated annual percentage change (EAPC) in prostate cancer burden and the Sociodemographic Index (SDI). By integrating SDI with the APC model, we analyze the changes in prostate cancer disease burden based on SDI across three levels: age, period, and birth cohort. Lastly, we employ the BAPC model to forecast the global trends in prostate cancer disease burden from 2021 to 2040.

## Methods

### Data source

The Global Burden of Disease (GBD) 2021 study comprehensively assessed health losses attributable to 371 diseases, injuries, and conditions, as well as 88 risk factors across 204 countries and regions, using the latest epidemiological data. For this study, we extracted data on the incidence, mortality, and disability-adjusted life years (DALYs) for prostate cancer from the publicly accessible GBD 2021 dataset. These data include global, continental, and national estimates. As prostate cancer is more prevalent among middle-aged and older men, this study selected men aged 40 and above as the study population and applied direct standardization to age-standardize the disease burden for this group. Age-standardized rates help eliminate the influence of age structure differences, making it suitable for fair comparisons between countries, regions, and populations. The study adheres to the Guidelines for Accurate and Transparent Health Estimates Reporting.

### Epidemiological trends and annual percentage change

We analyzed the relationship between the burden of prostate cancer and the Sociodemographic Index (SDI) in each region to explore the association between disease burden and societal development. We calculated the estimated annual percentage change (EAPC) for the age-standardized incidence, mortality, and DALY rates of prostate cancer corresponding to different SDI values, and conducted a spearman correlation analysis between EAPC and SDI.

### Age-Period-Cohort (APC) model analysis

The Age-Period-Cohort (APC) model is widely regarded as an advanced method that goes beyond traditional analyses in health and social sciences, helping to reveal the contributions of historical technological innovations, social changes, and health behaviors related to early life to disease trends [11, 12].

In APC model analysis, age groups are typically defined in 5-year intervals, corresponding to 5-year periods. Therefore, this study incorporated data from the 2021 Global Burden of Disease (GBD) database, covering the incidence, mortality, and DALY rates of prostate cancer among men aged 40 and above over the past 30 years (1992–2021), along with corresponding regional population data. The study population was divided into twelve age groups for further assessment: 40–44, 45–49, 50–54, and so on, up to 90–94 and 95+. The time periods from 1992 to 2021 were then categorized into six 5-year intervals: 1992–1996, 1997–2001, 2002–2006, 2007–2011, 2012–2016, and 2017–2021. Additionally, we analyzed seventeen overlapping 10-year birth cohorts, ranging from 1892 to 1901 to 1972–1981. For the three indicators—incidence, mortality, and DALY rates—each analysis included six regions: High SDI, High-middle SDI, Middle SDI, Low-middle SDI, Low SDI, and Global.

For example, in global incidence, the APC model estimates the overall time trend as well as the trends within specific age groups. The former is represented by the annual percentage change in incidence, referred to as net drift (annual percentage change), which is determined by both calendar time and continuous birth cohorts; the latter represents the annual incidence change by age, referred to as local drift (annual percentage change). Even slight changes in drift (annual percentage) can significantly alter the fitting rate over a 30-year period. The significance of annual percentage change trends was assessed using the Wald χ2 test. In the APC model, the age effect is described by age-specific incidence rates that are consistent with birth cohorts, while the period/cohort effect is represented by the relative risk of incidence associated with the period/cohort, calculated by comparing the age-specific incidence rates of each period/cohort with those of the reference period/cohort. The choice of the reference period/cohort is arbitrary and does not affect the interpretation of the results. All analyses and visualizations were performed using R (V.4.2.1).

### Bayesian Age-Period-Cohort (BAPC) model prediction

This study employs the Bayesian Age-Period-Cohort (BAPC) model, which integrates nested Laplace approximations, to forecast the global prostate cancer disease burden (including incidence, mortality, DALY rates, and corresponding case numbers) from 2022 to 2040 [13].

## Results

### Epidemiological changes in prostate Cancer

**Fig. 1:**
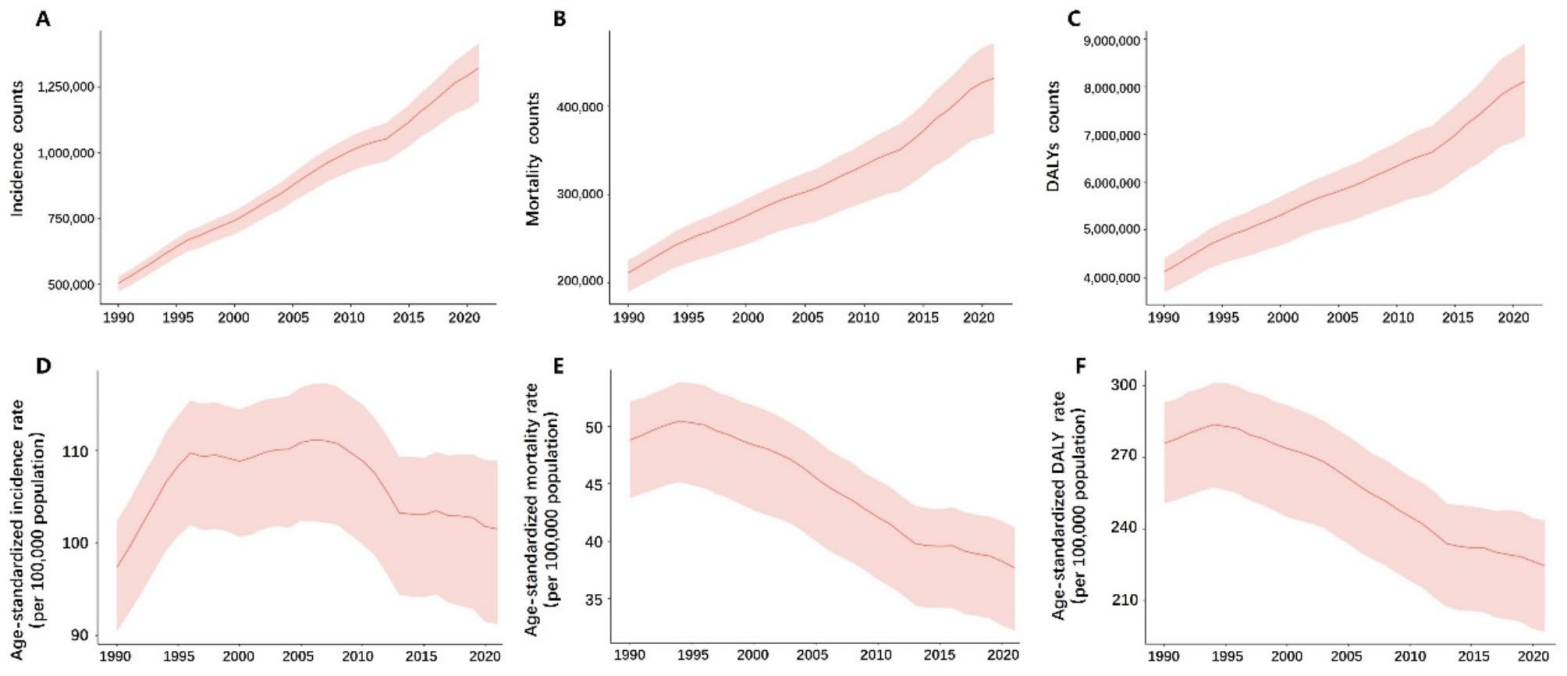
Trends of Prostate Cancer from 1990 to 2021: (**A**) Incidence, (**B**) Mortality, (**C**) DALY counts; and Age-standardized (**D**) Incidence rate, (**E**) Mortality rate, (**F**) DALY rate

From 1990 to 2021, the absolute number of prostate cancer cases, mortality, and disability-adjusted life years (DALYs) has shown a steady increase (Fig. 1A-C). However, the age-standardized rates for these indicators generally exhibited an initial rise followed by a subsequent decline. By 2021, both the age-standardized mortality rate and DALY rate for prostate cancer were significantly lower than in 1990, while the age-standardized incidence rate (ASIR) remained higher than its 1990 level (Fig. 1D-F) (Supplementary Table S1). In 2021, the global ASIR of prostate cancer was 15.37 per 100,000, an increase from 13.69 per 100,000 in 1990. However, both the age-standardized mortality rate (5.26 per 100,000) and the DALYs rate (95.94 per 100,000) showed a marked decrease compared to the 1990 rates of 6.33 per 100,000 and 113.81 per 100,000, respectively (Table 1).

**Table 1:** Global burden of prostate cancer in 1990 and 2021

Location	1990 age- standardized incidence rate (95% UI)	1990 age- standardized mortality rate (95% UI)	1990 age- standardized DALYs rate (95% UI)	2021 age- standardized incidence rate (95% UI)	2021 age- standardized mortality rate (95% UI)	2021 age- standardized DALYs rate (95% UI)
Global	13.69 (12.95–14.18)	6.33 (5.81–6.68)	113.81 (103.92-120.75)	15.37 (14.13–16.25)	5.26 (4.64–5.64)	95.94 (84.62-103.71)
Andean Latin America	12.36 (10.05–15.29)	10.64 (8.79-13)	178.41 (144.71-221.43)	19.73 (14.38–27.08)	10.14 (7.54–13.47)	173.29 (127.84–232.9)
Australasia	45.72 (41.65–48.83)	15.29 (13.88–16.15)	290.72 (264.67-308.31)	46.22 (39.03–54.5)	8.6 (7.31–9.86)	161.8 (136.7-188.2)
Caribbean	28.48 (26.32–31.53)	16.11 (14.83–18.08)	279.02 (257.36-314.91)	43.08 (36.88–49.62)	17.03 (14.68–19.85)	310.36 (265.37-364.87)
Central Asia	4.08 (3.8–4.33)	2.91 (2.71–3.1)	62.34 (58.27–66.27)	6.08 (5.49–6.73)	3.49 (3.18–3.84)	70.94 (64.26–78.44)
Central Europe	9.7 (9.19–10.27)	6.58 (6.24–7.01)	122.81 (116.51-130.06)	20.38 (18.49–22.4)	8.13 (7.44–8.78)	154.2 (139.8-167.22)
Central Latin America	17.19 (16.3-17.89)	9.17 (8.6–9.55)	161.57 (153.65-168.18)	29.51 (25.44–33.84)	8.59 (7.49–9.68)	160.68 (139.21-183.77)
Central Sub-Saharan Africa	9.96 (6.49–13.44)	10.71 (6.91–14.73)	182.87 (119.47-246.03)	11.18 (6.83–15.41)	10.51 (6.31–14.59)	186.63 (112.46-256.04)
East Asia	2.1 (1.6–2.67)	1.85 (1.43–2.45)	31.96 (23.97–40.54)	4.46 (3.3–5.93)	2.04 (1.55–2.71)	34.81 (26.31–46.61)
Eastern Europe	7.2 (6.82–7.54)	3.4 (3.21–3.54)	75.04 (70.78–78.74)	20.73 (18.57–22.74)	6.04 (5.43–6.69)	132.07 (117.75–147.8)
Eastern Sub-Saharan Africa	8.88 (5.39–11.3)	8.97 (5.46–11.38)	168.51 (101.48-214.68)	10.28 (6.65–12.89)	8.92 (5.81–11.25)	170.85 (109.53-217.87)
High-income Asia Pacific	5.6 (5.23–5.92)	2.61 (2.43–2.75)	46.42 (43.86–49.08)	12.67 (11.14–14.04)	3.04 (2.73–3.26)	54.95 (49.39–59.59)
High-income North America	54.44 (52.21–56.17)	11.04 (10.37–11.46)	229.69 (216.39-243.55)	47.02 (44.47–49.04)	6.89 (6.22–7.28)	145.47 (133.31–157.2)
North Africa and Middle East	5.99 (4.47–7.57)	4.31 (3.18–5.55)	73.49 (54.94–92.85)	13.64 (9.6-16.61)	4.95 (3.49-6)	86.04 (61.03-103.68)
Oceania	9.76 (6.8–13.3)	9.26 (6.35–12.75)	157.45 (108.38-216.43)	12.31 (7.98–17.2)	10.71 (6.8–15.1)	182.26 (115.59-257.96)
South Asia	2.64 (1.86–3.25)	2.64 (1.89–3.29)	47.71 (33.62–58.55)	3.6 (2.96–4.94)	2.79 (2.3–3.81)	49.52 (40.39–67.98)
Southeast Asia	4.14 (2.89–4.86)	3.66 (2.58–4.34)	65.82 (45.76–77.34)	7.27 (4.78-9)	4.54 (2.96–5.57)	82.97 (54.53-100.89)
Southern Latin America	13.84 (12.35–15.42)	10.71 (9.66–11.9)	193.07 (174.61-215.03)	18.75 (16.04–21.7)	9.18 (7.91–10.41)	164.37 (141.57-188.86)
Southern Sub-Saharan Africa	14.53 (10.53–19.28)	13.34 (9.83–17.55)	240.11 (174.65-319.46)	20.77 (15.38–24.47)	15.65 (11.28–18.31)	293.94 (214.73-345.64)
Tropical Latin America	12.79 (12-13.42)	9.33 (8.73–9.8)	167.74 (158.29-175.13)	17.92 (16.69-19)	8.9 (8.17–9.47)	161.4 (150.26-171.63)
Western Europe	23.84 (22.73–24.77)	10.28 (9.74–10.71)	187.3 (178.78-195.14)	33.37 (30.4-35.82)	7.47 (6.68–8.04)	139.37 (125.92-151.62)
Western Sub-Saharan Africa	13.55 (7.92–17.67)	13.88 (8.14–18.07)	247.37 (143.09-324.38)	19.4 (10.16–25.81)	17.93 (9.48–23.46)	303.04 (160.87-403.38)

UI: Uncertainty interval; DALYs: Disability-adjusted life-years; The age-standardized incidence rate, age-standardized mortality rate, age-standardized DALYs rate are shown per 100,000 person-years

### Age trends in prostate Cancer

**Fig. 2:**
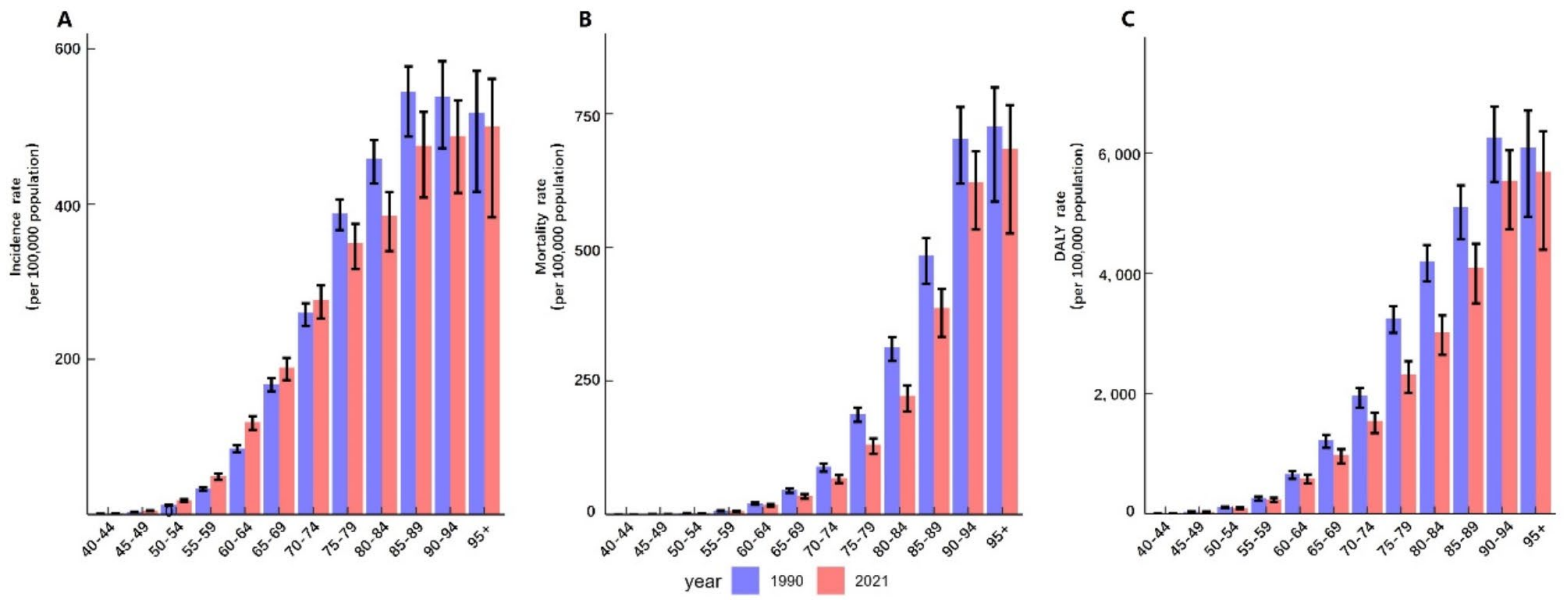
Prostate Cancer Rates in 1990 and 2021 by Age Group: (**A**) Incidence rate, (**B**) Mortality rate, (**C**) DALY rate

The incidence, mortality, and DALY rates of prostate cancer all increase with age. In 2021, mortality and DALY rates for all age groups were lower compared to 1990 (Fig. 2B-C). However, the incidence rate of prostate cancer in 2021 was higher than in 1990 for age groups under 75 years old (Fig. 2A) (Supplementary Table S2).

### Geographical trends in prostate Cancer

Prostate cancer burden shows significant geographic variation.

**Fig. 3:**
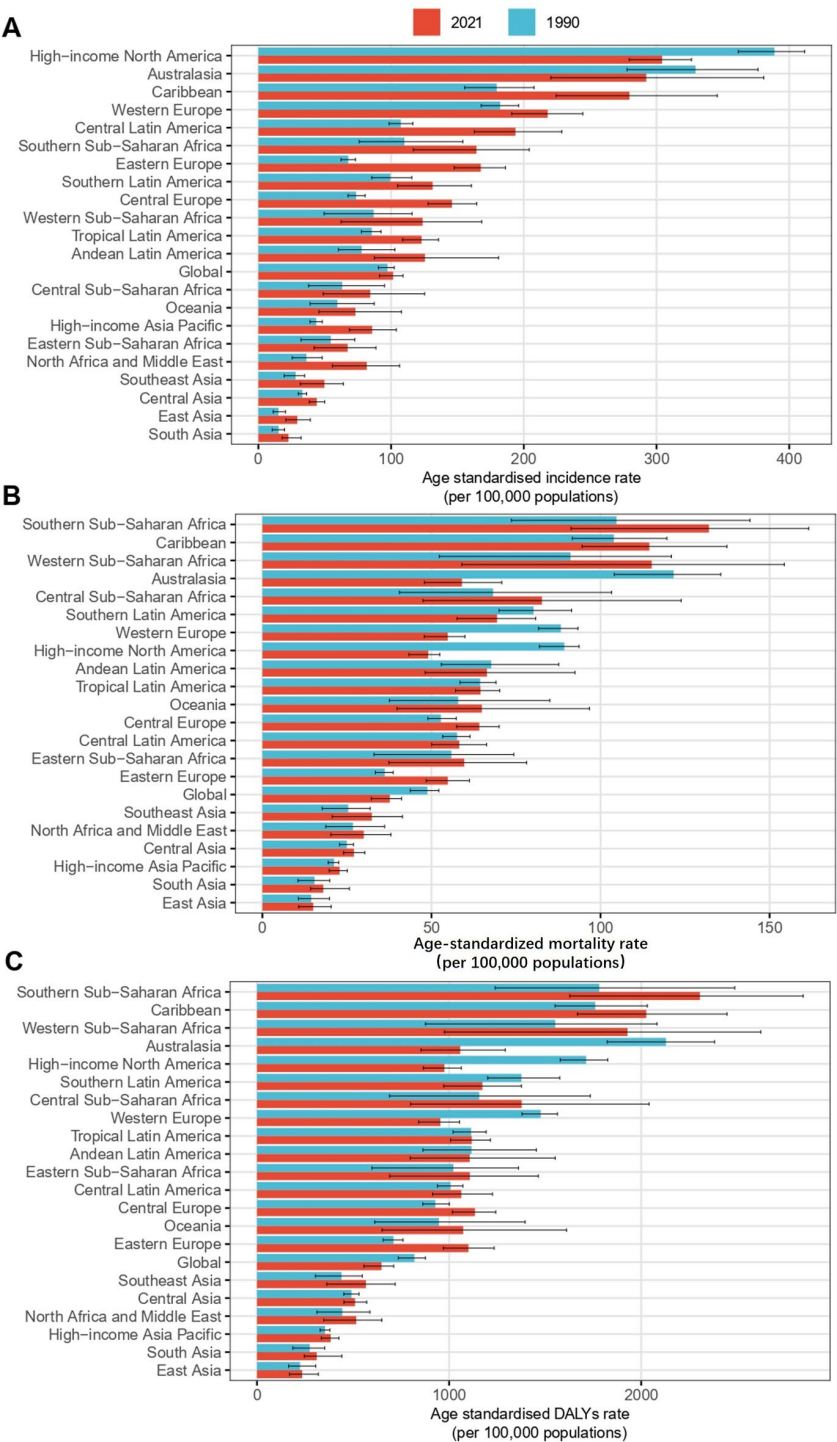
Age-standardized rates of prostate cancer by region in 1990 and 2021: (**A**) Incidence, (**B**) Mortality, (**C**) DALYs

**Fig. 4:**
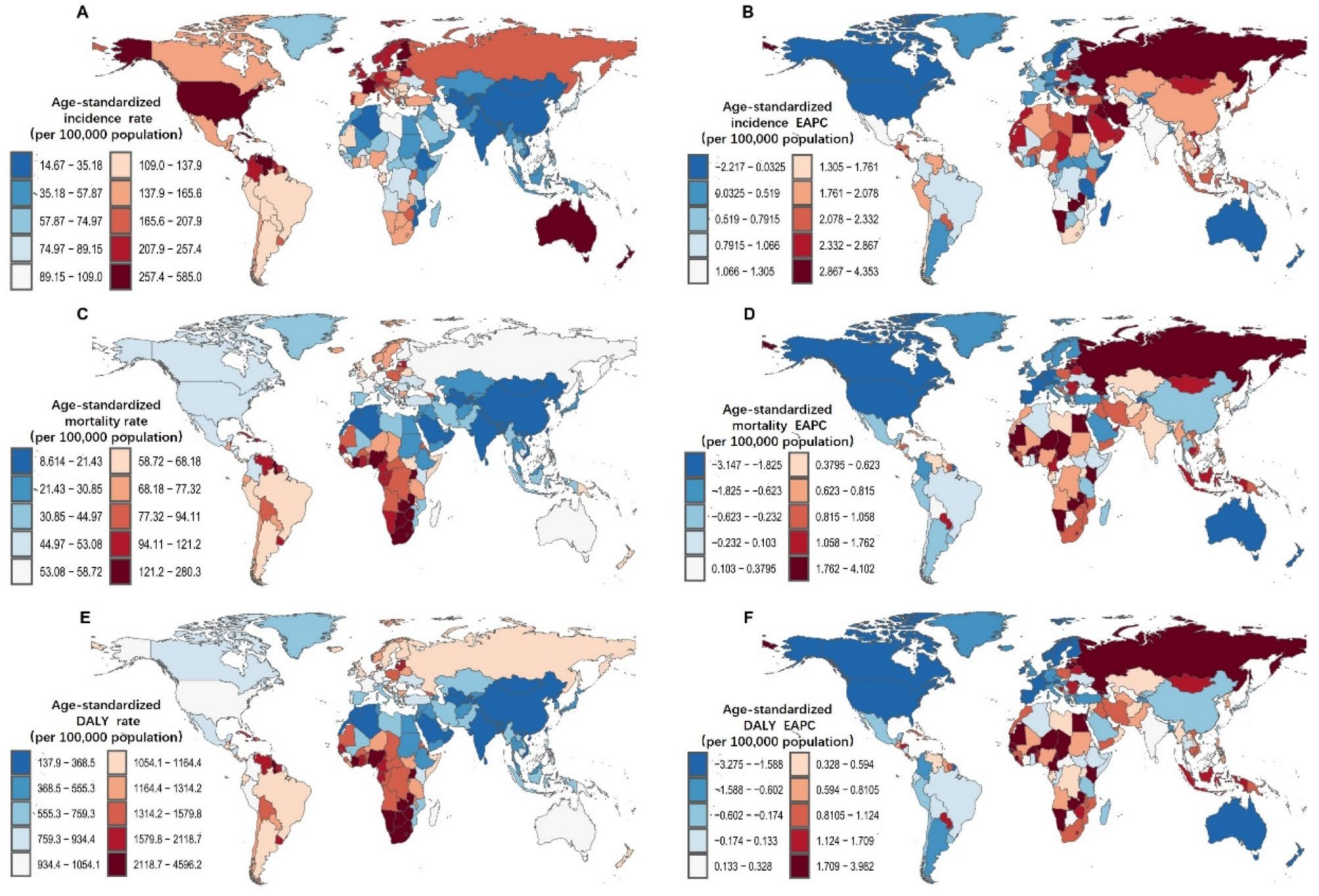
Age-standardized rates of prostate cancer by country in 1990 and 2021: (**A**) Incidence, (**B**) Mortality, (**C**) DALYs

In 2021, the highest age-standardized incidence rates (ASIR) were observed in High-income North America and Australasia among the 21 GBD regions, with High-income North America (304.23/100,000; 95% CI: 279.47-326.35) and Australasia (292.27/100,000; 95% CI: 220.36-380.77). Although the incidence rates in these regions remain high, they have decreased by 22% and 11%, respectively, compared to 1990. In contrast, the ASIR in all other regions showed an upward trend, with the highest increases observed in Eastern Europe (154%) and North Africa and the Middle East (121%). The regions with the lowest ASIR were South Asia (20.68/100,000; 95% CI: 17.83–32.24) and East Asia (29.31/100,000; 95% CI: 20.74–39.13) (Fig. 3A). At the country and territory level, the highest ASIR were observed in Bermuda (584.98; 95% CI: 405.13-828.26) and Antigua and Barbuda (506.14; 95% CI: 370.72-668.21). The lowest rates were found in the Republic of Tajikistan (14.66; 95% CI: 8.54–24.91) and Mongolia (17.84; 95% CI: 11.1-26.95). The countries with the highest EAPC (estimated annual percentage change) were Georgia (4.353; 95% CI: 3.594–5.118) and the Republic of Korea (4.352; 95% CI: 3.741–4.967) (Fig. 4A). Canada and the Republic of Tajikistan had the lowest EAPC, at -2.217 (95% CI: -2.573, -1.859) and − 1.310 (95% CI: -1.492, -1.128), respectively (Fig. 4B).

Regarding age-standardized mortality rates (ASMR), in 2021, the highest rates were found in Southern Sub-Saharan Africa (132.06/100,000; 95% CI: 91.28-161.54) and Western Sub-Saharan Africa (115.14/100,000; 95% CI: 58.97-154.33). Both of these regions saw an increase in ASMR rates by 26% compared to 1990, following Eastern Europe’s 51% and Southeast Asia’s 27%. The regions with the lowest ASMR were still East Asia (15.04/100,000; 95% CI: 10.73–20.32) and South Asia (18.00/100,000; 95% CI: 14.22–25.73). The fastest declines in mortality rates were seen in Australasia and High-income North America, with decreases of 51% and 45%, respectively (Fig. 3B). Among the countries, Grenada and Saint Kitts and Nevis had the highest ASIRs, at 280.29 (95% CI: 220.3-349.97) and 280.2 (95% CI: 210.81-363.41), respectively. The lowest ASIRs were found in the People’s Democratic Republic of Algeria (8.61; 95% CI: 4.6-13.95) and the Republic of Tajikistan (11.09; 95% CI: 6.54–19.25) (Fig. 4C). The countries with the highest ASIR EAPC were Georgia (4.102; 95% CI: 3.204–5.009) and the Arab Republic of Egypt (2.585; 95% CI: 2.240–2.932). The lowest ASIR EAPCs were observed in Canada (-3.147; 95% CI: -3.373, -2.921) and Australia (-2.897; 95% CI: -3.280, -2.513) (Fig. 4D).

In 2021, the highest age-standardized DALY rates were found in Southern Sub-Saharan Africa (2305.79/100,000; 95% CI: 1628.21-2843.45) and the Caribbean (2026.32/100,000; 95% CI: 1668.43-2447.11). Southern Sub-Saharan Africa’s age-standardized DALYs increased by 29%, second only to Eastern Europe’s 54%. The regions with the lowest age-standardized DALY rates were still East Asia (235.13/100,000; 95% CI: 168.83-319.68) and South Asia (310.90/100,000; 95% CI: 246.61-442.17). The fastest declines in DALY rates were observed in Australasia and High-income North America, with decreases of 50% and 43%, respectively (Fig. 3C). Interestingly, the highest age-standardized DALYs were found in Georgia (4596.17; 95% CI: 3593.57-5754.12) and the Arab Republic of Egypt (4523.25; 95% CI: 3331.66-5961.62), while the lowest were in the People’s Democratic Republic of Algeria (137.91; 95% CI: 76.68-220.68) and the Socialist Republic of Vietnam (191.51; 95% CI: 93.37-308.27) (Fig. 4E). Regarding EAPC, the highest were observed in Georgia (3.982; 95% CI: 3.183–4.788) and Zambia (2.578; 95% CI: 2.228–2.928); the lowest were again in Canada (-3.275; 95% CI: -3.552, -2.998) and Australia (-2.802; 95% CI: -3.263, -2.339) (Fig. 4F) (Table 1) (Supplementary Table S3).

**Fig. 5:**
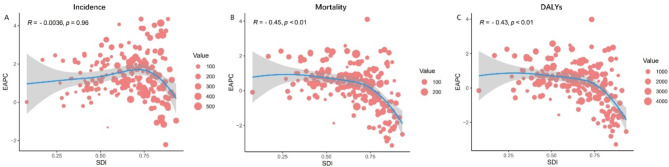
Correlation analysis of the Sociodemographic Index (SDI) with the estimated annual percentage change (EAPC) in age-standardized (**A**) incidence rate, (**B**) mortality rate, and (**C**) DALY rate of prostate cancer in 2021

The regions were classified according to the Socio-Demographic Index (SDI), and a correlation analysis was conducted between the estimated annual percentage change (EAPC) of age-standardized incidence rates, mortality rates, and DALY rates. The results showed that the EAPC of age-standardized incidence rates had no correlation with SDI (Fig. 5A), while the EAPC of mortality rates and DALY rates showed a negative correlation with SDI (*p* < 0.01) (Fig. 5B-C) (Supplementary Table S3).

### Age-Period-Cohort (APC) model analysis

**Fig. 6:**
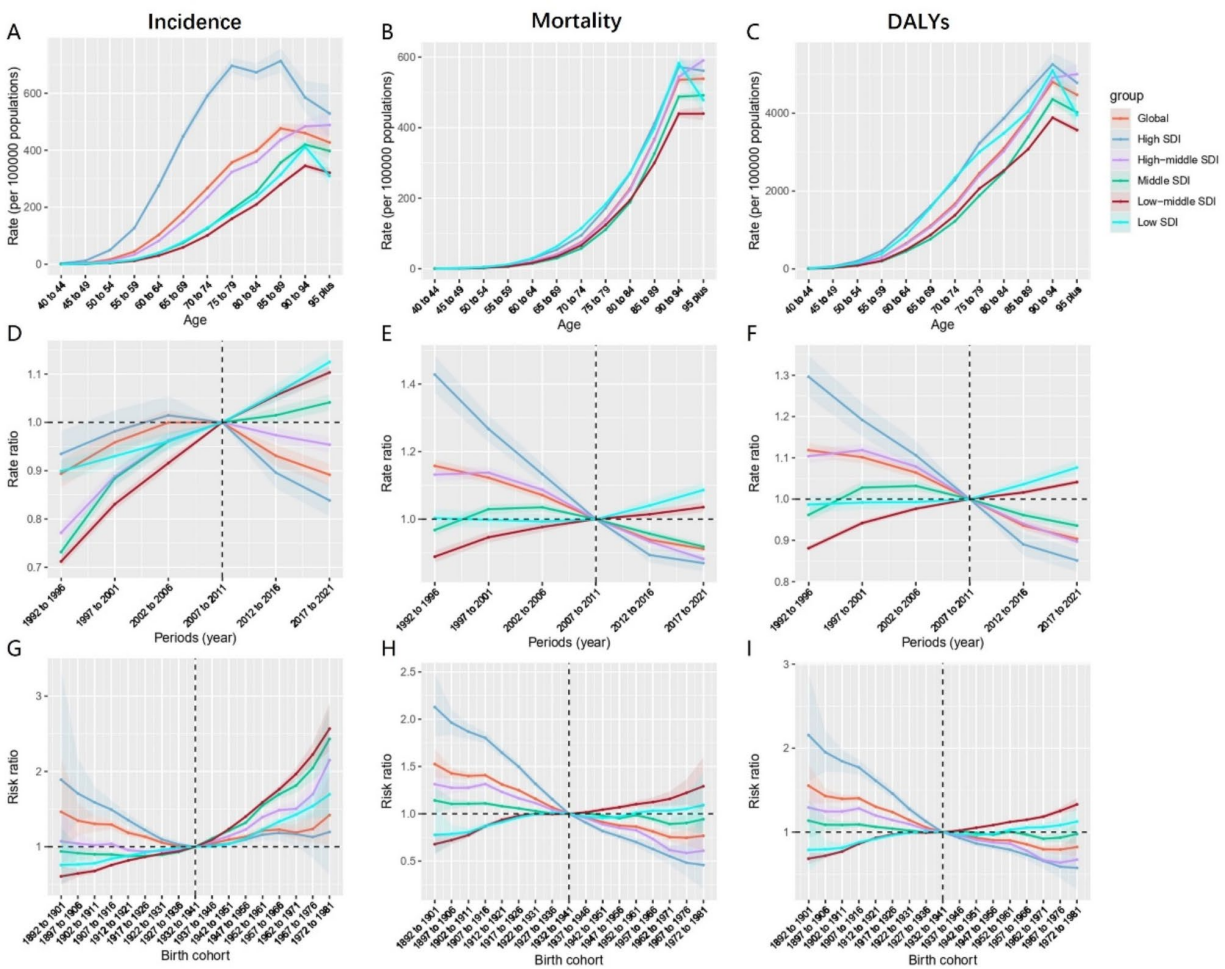
Age Trends of Prostate Cancer Based on SDI: (**A**) Incidence rate, (**B**) Mortality rate, (**C**) DALY rate; Age-standardized Trends by Period: (**D**) Incidence rate, (**E**) Mortality rate, (**F**) DALY rate; Age-standardized Trends by Birth Cohort: (**G**) Incidence rate, (**H**) Mortality rate, (**I**) DALY rate

At the age level, except for the High-middle SDI regions, the incidence rates in other regions showed an upward trend followed by a decline with increasing age. Among these, the incidence rates in the High SDI regions were significantly higher than in other regions at all ages. As for mortality, except in the High-middle SDI regions where mortality continued to rise with age, both the High SDI and Low SDI regions showed an initial increase in mortality with age, followed by a decrease in the population aged 95 and older. In contrast, mortality in the remaining regions—Global, Middle SDI, and Low-middle SDI—continued to rise with age, leveling off after age 95. Regarding DALY rates, except for the High-middle SDI regions where the rate continuously increased with age, all other regions showed an increase in DALY rates with age, followed by a decrease in the population aged 95 and older (Fig. 6A-C) (Supplementary Tables S4–S6).

At the period level, the incidence of prostate cancer in the Global, High SDI, and High-middle SDI regions initially increased and then declined. The key difference was that the peak incidence in the High SDI regions occurred in the 2002–2006 period, while the peak in the High-middle SDI regions was in the 2007–2011 period, and the Global region remained near its peak from 2002 to 2011. The incidence in the remaining regions continued to rise. As for mortality, only the Low-middle SDI and Low SDI regions showed an overall increasing trend, while all other regions exhibited an overall downward trend, with the High SDI regions showing the fastest decline and the Middle SDI regions the slowest. DALY rates followed a trend similar to that of mortality (Fig. 6D-F) (Supplementary Tables S7–S9).

At the birth cohort level, the incidence rates in the Low-middle SDI and Low SDI regions showed a consistent upward trend, with the increase being faster in the Low-middle SDI regions. In contrast, the incidence in other regions exhibited an initial decline followed by an increase. Regarding mortality, except for the Low-middle SDI and Low SDI regions where an overall increase was observed, all other regions showed a general decline, with the High SDI regions decreasing the most rapidly. Similarly, DALY rates exhibited trends similar to those of mortality (Fig. 6G-I) (Supplementary Tables S10–S12).

### Global prostate Cancer burden prediction to 2040

**Fig. 7:**
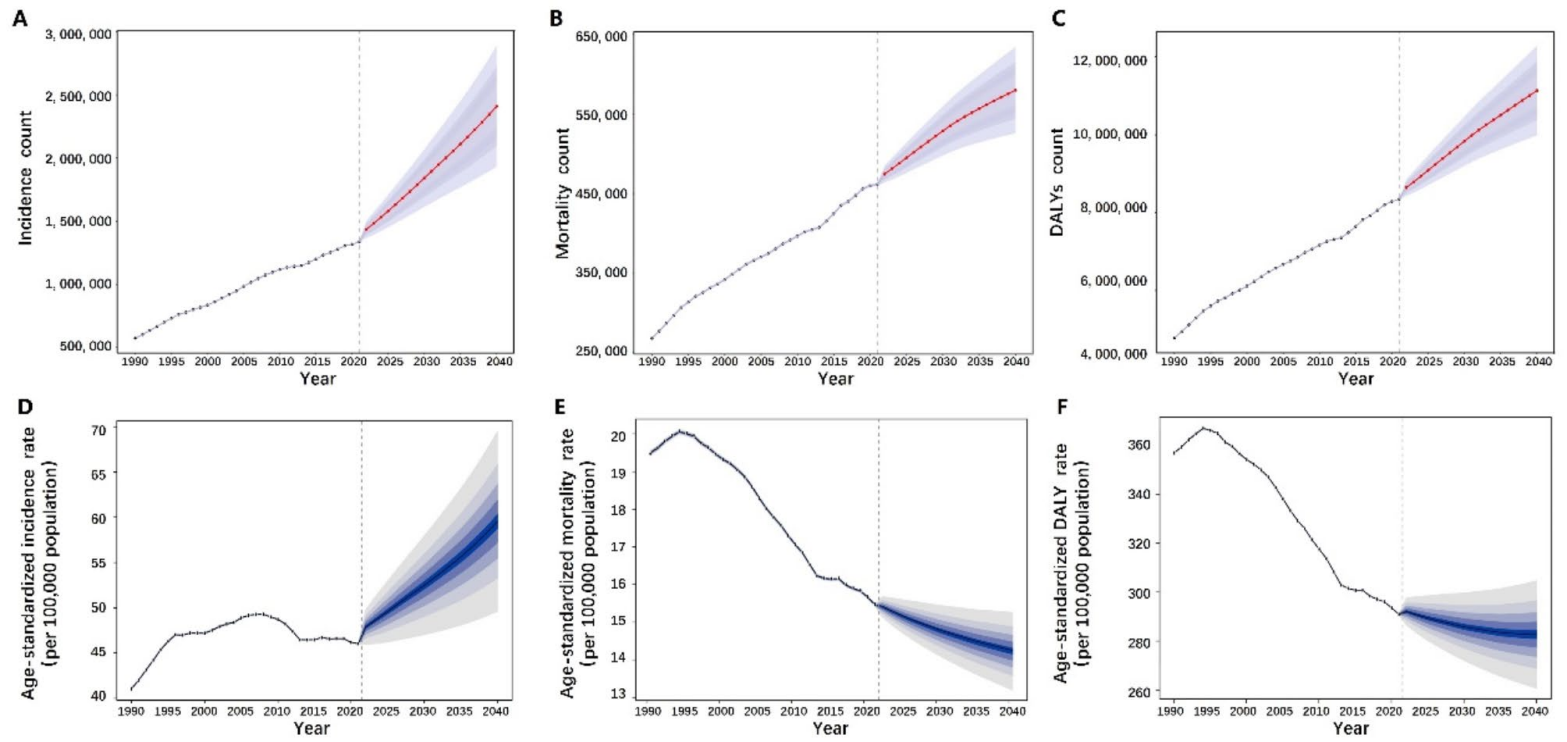
Trends and Predictions of Prostate Cancer from 1990 to 2040: (**A**) Incidence, (**B**) Mortality, (**C**) DALY values; Age-standardized Trends and Predictions: (**D**) Incidence rate, (**E**) Mortality rate, (**F**) DALY rate

It is predicted that from 2021 onwards, the total number of prostate cancer cases globally will continue the increasing trend observed from 1990 to 2021, reaching 2,408,776.03 cases by 2040 (95% UI: 1,929,826.28-2,887,725.80) (Fig. 7A). Similarly, the number of prostate cancer mortality and DALYs will also continue to increase, reaching 579,684.54 mortality (95% UI: 525,380.27–633,988.81) and 11,136,530.21 DALYs (95% UI: 9,982,972.82-12,290,087.60) by 2040 (Fig. 7B-C).

Regarding age-standardized rates, interestingly, although the incidence of prostate cancer has shown a slow overall decline over the past decade, predictive analysis indicates that it will exhibit a rapid upward trend after 2021 (Fig. 7D). In contrast, mortality and DALY rates will generally continue the downward trend observed over the past 30 years (Fig. 7E-F) (Supplementary Table S13).

## Discussion

In 2022, there were 1.5 million new cases and 397,000 mortality from prostate cancer globally [2]. Although the global incidence rate, mortality rate, and DALY rate of prostate cancer have shown a declining trend in recent years, the number of new cases, deaths, and DALYs continues to rise due to global population growth and aging [8]. Moreover, it is foreseeable that these numbers will continue to increase in the future. Additionally, predictions based on the BAPC model indicate that from 2021 to 2040, the global prostate cancer incidence will rise counter to the general trend. Moreover, there are significant differences in the disease burden of prostate cancer across different regions and populations [14], all of which present challenges for reducing the burden of this disease. Therefore, this study utilized the most recent 2021 global, regional, and national data from the Global Burden of Disease (GBD) study to analyze the disease burden of prostate cancer, aiming to provide theoretical support for mitigating this major public health burden.

The positive correlation between prostate cancer incidence and age has been confirmed by numerous studies [15, 16], and our study is no exception. Additionally, we found significant improvements in the disease burden of prostate cancer in 2021 compared to 1990, in terms of incidence, mortality, and DALYs. The only exception was in men younger than 75, where the prostate cancer incidence in 2021 was higher than in 1990. This may be related to the fact that recent guidelines have recommended initiating prostate cancer screening at age 55 or even earlier [17–19], which could also explain why prostate cancer incidence is expected to continue rising in the future. While the necessity of early prostate cancer screening remains a subject of debate in the academic community [20, 21], it is unclear at this point how the increased incidence in these populations will impact mortality and DALY rates.

In 2021, high-income regions such as North America and Australia had the highest age-standardized prostate cancer incidence rates, although these rates have significantly decreased compared to 1990. In contrast, regions such as sub-Saharan Africa, Western and Caribbean countries exhibited the highest ASMR and DALY rates for prostate cancer, and these trends continue to rise. It is widely believed that the widespread introduction of PSA screening in developed countries in the late 1980s is the primary cause of the sharp increase in prostate cancer incidence in the following decade [22]. This was followed by innovations in treatment modalities, such as radical prostatectomy [23], radiotherapy [24], and androgen deprivation therapy [25], which significantly reduced age-standardized mortality and DALY rates in high-income regions. This also explains why the estimated annual percentage change (EAPC) in age-standardized prostate cancer mortality and DALY rates is negatively correlated with the Socio-Demographic Index (SDI). Of course, low-income countries lag far behind high-income countries in terms of the timing and coverage of prostate cancer screening and treatment methods [26, 27]. Furthermore, the higher susceptibility of Black populations to prostate cancer is one of the contributing factors to the heavier disease burden in underdeveloped regions of Africa [28, 29].

In the Age-Period-Cohort (APC) model analysis, at the age level, the incidence rate of prostate cancer in High SDI regions is significantly higher across all age groups compared to other regions, while mortality and DALY rates do not show such a pattern. This could be attributed to the widespread implementation of prostate cancer screening in these regions at an early stage.

At the period level, the incidence of prostate cancer in High SDI regions steadily increased between 1992 and 2001. However, from 2002 to 2006, it began to decline, with a more rapid decrease observed between 2007 and 2011. This change may partly be due to the US Preventive Services Task Force (USPSTF) recommendation in 2008 against PSA screening for men aged 75 and older [30], and in 2012, a broader recommendation against PSA screening for all age groups due to concerns about over-diagnosis and benefit-to-harm ratios [31]. Following these updates, the willingness of both doctors and the general population in Canada and Australia to conduct PSA tests also declined gradually [32, 33]. Whether similar trends in High-middle SDI and Global regions are also due to similar reasons remains unclear. In contrast, the incidence of prostate cancer in Middle SDI and lower SDI regions has steadily increased over time, which may be due to the more recent introduction and gradual widespread adoption of PSA screening. Regarding mortality and DALYs, the trends in Low-middle SDI and Low SDI regions show a continuous increase over time, while other regions exhibit a general decline. The rate of decline is roughly positively correlated with SDI, which aligns with our hypothesis—that regions with higher SDI provide more adequate treatment for prostate cancer, resulting in lower mortality and DALYs.

As for the cohort analysis, the trends in mortality and DALYs across different birth cohorts are similar to those observed across periods. This can be attributed to the gradual spread of prostate cancer screening in Low-middle SDI and Low SDI regions, combined with increasing life expectancy. These regions are experiencing a gradual increase in prostate cancer incidence. However, the development of treatment conditions is much slower, leading to higher mortality and DALYs in younger birth cohorts. As for incidence, Low-middle SDI and Low SDI regions continue to see increases across birth cohorts, whereas other regions exhibit a trend of initial decline followed by an increase. Interestingly, we found that in these regions, the higher the SDI, the faster the incidence decline in earlier birth cohorts and the slower the increase in later cohorts. We hypothesize that higher SDI regions implemented early prostate cancer screening earlier, leading to a rapid decline in incidence in earlier cohorts as a result of detecting many prostate cancer cases in the short term. This explains the subsequent slow recovery in the incidence rate in later birth cohorts. The gradual increase in prostate cancer incidence in Low-middle SDI and Low SDI regions across birth cohorts may reflect the delayed implementation of screening in these regions compared to High SDI areas.

Compared with existing studies on the global disease burden of prostate cancer, this study has the following advantages. First, it is the first to select men aged 40 and above as the study population and to apply age-standardization. This approach helps eliminate biases caused by differences in age structure across countries or regions. In addition, this study is the first to use the Age-Period-Cohort (APC) model in conjunction with the Socio-Demographic Index (SDI) for stratified analysis. The disease burden of prostate cancer was analyzed from four perspectives: age, period, cohort, and SDI. This multi-dimensional approach provides a more comprehensive representation of the findings. Finally, the study also predicts the trends of prostate cancer disease burden from 2022 to 2040, offering public health policymakers a degree of foresight regarding future developments.

Nevertheless, this study has limitations. First, the healthcare levels in some underdeveloped countries may lead to misdiagnosis and missed diagnoses, resulting in an underestimation of DALYs. Second, the GBD collaborators used extensive statistical modeling methods, especially at the national level, with the data heavily reliant on modeling due to a lack of original data. Third, the absence of sub-national data limits the ability to study disease trends at a sub-national level. Finally, the lag in GBD data is also a concern.

Prostate cancer is a major public health issue worldwide, but the differences between regions and populations are significant. Applying the same healthcare policies universally may not be effective. Despite notable achievements in reducing the disease burden from 1990 to 2021, there is still a long way to go to achieve low disease burdens globally. Addressing the prostate cancer burden requires comprehensive intervention measures, prioritizing high-risk groups. High-income regions such as North America and Australasia should continue to invest in prostate cancer diagnosis and treatment to reduce the disease burden to levels appropriate for their level of societal development. Conversely, Sub-Saharan Africa, Western Africa, and the Caribbean need to vigorously promote prostate cancer screening and treatment to curb the rising disease burden. Targeted diagnostic and treatment plans should be developed for different populations. The balance between the economic and health benefits of early PSA screening requires further research to provide stronger evidence for its effectiveness.

## Electronic supplementary material

Below is the link to the electronic supplementary material.

Supplementary Material 1

## Data Availability

The data used in this study are freely available for download from the GBD 2021 website (https://vizhub.healthdata.org/gbd-results/).
