# Supplementary material for: Global burden of prostate cancer: age-period-cohort analysis from 1990 to 2021 and projections until 2040
Source: World J Surg Oncol. 2025 Mar 20;23:98. doi: 10.1186/s12957-025-03733-1 (PMC11924780; doi:10.1186/s12957-025-03733-1)
Supplement: Supplementary file 1 — Supplementary Material 1 [file 12957_2025_3733_MOESM1_ESM.docx]

Supplementary table 1 Global counts and ASR of prostate cancer incidence, mortality, and DALYs, 1990-2021.

| Year | Incidence | | Mortality | | DALYs | |
| --- | --- | --- | --- | --- | --- | --- |
|  | Counts  (95% CI) | Rate  (95% CI) | Counts  (95% CI) | Rate  (95% CI) | Counts  (95% CI) | Rate  (95% CI) |
| 1990 | 505157.07  (471904.64 - 530213.59) | 97.38  (90.47 - 102.43) | 211536.13  (189983.98 - 225719.34) | 48.8  (43.74 - 52.16) | 4120724.39  (3691958.32 - 4407374.22) | 819.6  (735.53 - 876.77) |
| 1991 | 530205.88  (495828.02 - 555725.28) | 99.5  (92.51 - 104.52) | 219068.86  (196921.36 - 232845.93) | 49.21  (44.14 - 52.42) | 4259358.11  (3815060.89 - 4543553) | 825.73  (740.84 - 881.07) |
| 1992 | 557735.92  (520948.3 - 584649.09) | 101.88  (94.61 - 107.01) | 227183.08  (203747.2 - 241339.19) | 49.72  (44.5 - 52.91) | 4409400.53  (3944886.78 - 4718567.34) | 833.47  (747.01 - 891.75) |
| 1993 | 585596.66  (548159.62 - 612785.87) | 104.17  (96.93 - 109.26) | 234986.22  (210960.18 - 249439.25) | 50.13  (44.91 - 53.33) | 4555044.83  (4078116.85 - 4860140.92) | 839.84  (753.35 - 896.17) |
| 1994 | 615601.26  (576368.09 - 644759.57) | 106.58  (99.21 - 111.92) | 242869.84  (217721.05 - 258602.86) | 50.45  (45.13 - 53.85) | 4703628.9  (4209530.74 - 5027627.44) | 845.55  (757.8 - 903.77) |
| 1995 | 643073.16  (601700.67 - 673664.65) | 108.34  (100.79 - 113.8) | 248595.72  (222132.63 - 264957.52) | 50.3  (44.88 - 53.73) | 4811377.77  (4294881.48 - 5155472.63) | 843.33  (753.96 - 903.78) |
| 1996 | 669487.81  (625711.33 - 702083.92) | 109.75  (101.99 - 115.38) | 254311.79  (226714.16 - 271051.02) | 50.12  (44.62 - 53.55) | 4917906.74  (4375841.52 - 5259376.41) | 840.26  (749.09 - 899.12) |
| 1997 | 685435.66  (638879.41 - 719322.36) | 109.34  (101.37 - 115.03) | 258724.78  (230268.35 - 275757.24) | 49.58  (44.09 - 52.97) | 4995405.24  (4437178.13 - 5349705.65) | 831.07  (739.62 - 890.39) |
| 1998 | 706209.89  (658245.33 - 741095.33) | 109.52  (101.54 - 115.2) | 264654.7  (235126.59 - 282107.64) | 49.24  (43.73 - 52.62) | 5106020.51  (4521112.39 - 5478672.1) | 826.36  (733.38 - 886.87) |
| 1999 | 724334.82  (675623.98 - 759366.43) | 109.19  (101.28 - 114.77) | 269881.24  (239254 - 287786.18) | 48.76  (43.18 - 52.12) | 5200034.47  (4600822.89 - 5572484.83) | 818.61  (725.5 - 877.61) |
| 2000 | 742475.44  (690732.59 - 778706.93) | 108.83  (100.69 - 114.45) | 275926.04  (243700.27 - 294782.52) | 48.36  (42.68 - 51.79) | 5305784.64  (4672040.86 - 5701041.55) | 812.26  (716.44 - 872.92) |
| 2001 | 767220.78  (712864.35 - 804807.9) | 109.26  (100.92 - 114.92) | 282450.17  (248669.62 - 301294.58) | 48.05  (42.28 - 51.39) | 5423536.97  (4774851.91 - 5815524.3) | 807.38  (711.72 - 866.09) |
| 2002 | 794301.86  (739339.22 - 833640.93) | 109.78  (101.58 - 115.49) | 288598.19  (254686.94 - 307822.65) | 47.6  (41.96 - 50.87) | 5541129.83  (4884742.48 - 5939299.48) | 801.22  (707.17 - 859.03) |
| 2003 | 819999.69  (763407.7 - 859648.96) | 110.07  (101.86 - 115.66) | 294288.63  (259826.79 - 313358.97) | 47.09  (41.52 - 50.25) | 5645727.69  (4984118.49 - 6051607.84) | 793.55  (701.22 - 850.66) |
| 2004 | 845006.13  (784375.8 - 887168.6) | 110.14  (101.66 - 115.88) | 298812.5  (263596.76 - 319233.81) | 46.36  (40.82 - 49.62) | 5728936.81  (5044471.4 - 6144448.34) | 782.5  (689.44 - 839.38) |
| 2005 | 876620.51  (814470.02 - 921883.5) | 110.87  (102.43 - 116.82) | 302942.85  (266740.74 - 324672.84) | 45.5  (40 - 48.83) | 5809998.19  (5120874.58 - 6252164.76) | 770.67  (679.4 - 829.15) |
| 2006 | 906648.83  (839468.28 - 954057.12) | 111.14  (102.35 - 117.17) | 307839.71  (270440.12 - 329525.79) | 44.72  (39.22 - 47.94) | 5894443.21  (5179546.71 - 6350433) | 758.51  (666.57 - 817.01) |
| 2007 | 934899.54  (865085.47 - 985542.15) | 111.05  (102.22 - 117.26) | 314025.83  (275730.16 - 336697.71) | 44.09  (38.64 - 47.35) | 5995719.81  (5268955.49 - 6450535.05) | 747.78  (657.18 - 804.63) |
| 2008 | 962308.53  (890749.03 - 1013665.03) | 110.74  (101.95 - 116.85) | 320908.06  (281505.22 - 344533.74) | 43.51  (38.1 - 46.76) | 6117092.33  (5373036.23 - 6595723.91) | 738.94  (649.12 - 796.45) |
| 2009 | 985201.93  (910359.01 - 1037936.37) | 109.79  (100.91 - 115.86) | 326854.13  (286712.19 - 350200.26) | 42.75  (37.43 - 45.87) | 6221014.77  (5458087.66 - 6713498.47) | 727.29  (637.95 - 784.39) |
| 2010 | 1007743.34  (929265.03 - 1062192.9) | 108.89  (99.88 - 114.96) | 333280.19  (290943.3 - 358697) | 42.07  (36.65 - 45.3) | 6333565.74  (5541952.52 - 6829601.96) | 717.22  (627.4 - 773.12) |
| 2011 | 1027063.11  (946758.23 - 1082076.03) | 107.56  (98.69 - 113.5) | 340264.97  (296132.65 - 366643.53) | 41.47  (36.05 - 44.69) | 6450681.65  (5638574.29 - 6977464.71) | 707.34  (618.05 - 764.67) |
| 2012 | 1041373.91  (958418.06 - 1099011.21) | 105.54  (96.69 - 111.55) | 345659.69  (301117.24 - 373446.89) | 40.59  (35.31 - 43.85) | 6543961.68  (5698108.01 - 7098492.46) | 693.61  (603.94 - 751.78) |
| 2013 | 1051614.51  (965966.3 - 1111175.38) | 103.28  (94.43 - 109.31) | 350743.17  (303713.83 - 379719.43) | 39.78  (34.39 - 43.06) | 6620989.83  (5765298.94 - 7168053.25) | 679.39  (591.25 - 735.31) |
| 2014 | 1084025.96  (995322.59 - 1146616.06) | 103.13  (94.25 - 109.29) | 360936.7  (312311.19 - 390612.97) | 39.61  (34.21 - 42.87) | 6797944.31  (5902192.51 - 7381487.89) | 675.68  (586.26 - 733.29) |
| 2015 | 1116439.77  (1024381.38 - 1180970.97) | 103.07  (94.16 - 109.22) | 372183.39  (322026.48 - 402257.18) | 39.56  (34.17 - 42.79) | 6984269.22  (6074343.91 - 7589367.31) | 673.57  (585.45 - 731.37) |
| 2016 | 1157166.38  (1061165.16 - 1225884.82) | 103.5  (94.51 - 109.83) | 385338.59  (332934.64 - 417648.05) | 39.6  (34.17 - 42.93) | 7212902.61  (6250717.7 - 7804879.97) | 673.66  (583.6 - 728.78) |
| 2017 | 1190053.61  (1086193 - 1262993.12) | 102.98  (93.6 - 109.46) | 394738.18  (339928.85 - 428751.47) | 39.12  (33.65 - 42.51) | 7394147.62  (6385405.07 - 8033795.31) | 667.43  (576.28 - 725.05) |
| 2018 | 1228495.19  (1117723.06 - 1306139.01) | 102.91  (93.24 - 109.58) | 406352.64  (350616.88 - 441925.06) | 38.88  (33.5 - 42.27) | 7605402.81  (6570966.38 - 8321466.22) | 664.08  (573.54 - 726.02) |
| 2019 | 1267076.08  (1149997.11 - 1349121.61) | 102.75  (92.88 - 109.55) | 419214.54  (360749.42 - 456346.76) | 38.7  (33.28 - 42.14) | 7830738.17  (6755171.65 - 8585439.88) | 661.35  (570.53 - 724.42) |
| 2020 | 1291414.63  (1165958.01 - 1380567.83) | 101.79  (91.54 - 108.96) | 426988.18  (364941.24 - 466249.36) | 38.24  (32.66 - 41.74) | 7982253.16  (6833549.93 - 8719621.28) | 654.84  (560.54 - 715.02) |
| 2021 | 1321834.97  (1192905.36 - 1416572.01) | 101.53  (91.28 - 108.96) | 431918.42  (369727.42 - 471621.95) | 37.68  (32.22 - 41.16) | 8108393.29  (6965891.44 - 8904316.4) | 648.28  (556.68 - 711.72) |

Abbreviations: ASR, age-standardized rate; CI, confidence interval; DALYs, disability adjusted life years.

Supplementary table 2 Global incidence, mortality, and DALY rates of prostate cancer in men aged 40 and above, 1990 and 2021.

| Age | Incidence | | Mortality | | DALYs | |
| --- | --- | --- | --- | --- | --- | --- |
|  | 1990  (95% UI) | 2021  (95% UI) | 1990  (95% UI) | 2021  (95% UI) | 1990  (95% UI) | 2021  (95% UI) |
| 40-44 | 0.88  (0.94 - 0.77) | 1.39  (1.53 - 1.16) | 0.22  (0.25 - 0.17) | 0.22  (0.25 - 0.16) | 11.41  (12.79 - 8.9) | 11.49  (13.06 - 8.73) |
| 45-49 | 3.4  (3.6 - 3.08) | 4.96  (5.4 - 4.41) | 0.81  (0.89 - 0.67) | 0.74  (0.84 - 0.59) | 37.32  (41.09 - 30.78) | 35.14  (40.46 - 28.6) |
| 50-54 | 12.32  (12.95 - 11.3) | 18.39  (19.8 - 16.52) | 2.67  (2.89 - 2.21) | 2.28  (2.56 - 1.84) | 110.26  (120.1 - 92.2) | 99.39  (112.18 - 81.89) |
| 55-59 | 33.35  (35.1 - 30.85) | 49.31  (52.57 - 44.57) | 7.23  (7.85 - 6.03) | 6.18  (6.95 - 5.04) | 264.41  (287.44 - 221.77) | 240.04  (267.16 - 198.18) |
| 60-64 | 85.23  (89.17 - 80.16) | 119.11  (126.72 - 109.15) | 20.96  (22.42 - 18.25) | 17.8  (19.58 - 14.93) | 662.84  (709.92 - 581.2) | 593.38  (653.29 - 507.25) |
| 65-69 | 167.89  (175.76 - 158.76) | 189.43  (201.92 - 173.24) | 45.84  (48.85 - 40.65) | 35  (38.18 - 29.61) | 1228.11  (1313.44 - 1098.91) | 976.26  (1074.65 - 839.68) |
| 70-74 | 260.07  (272.39 - 243.05) | 276.44  (295.3 - 252.87) | 89.63  (95.19 - 80.3) | 68.24  (73.83 - 58.9) | 1965.78  (2095.15 - 1764.47) | 1545.38  (1683.03 - 1342.28) |
| 75-79 | 387.95  (406.21 - 366.62) | 349.62  (374.7 - 316.35) | 187.71  (199.98 - 173.38) | 130.92  (142.44 - 113.38) | 3249.77  (3453.74 - 3011.13) | 2318.12  (2537.06 - 2010.95) |
| 80-84 | 458.62  (482.8 - 426.57) | 385.13  (415.78 - 339.63) | 312.7  (332.14 - 287.25) | 221.78  (241.71 - 192.92) | 4202.08  (4471.91 - 3864.57) | 3020.66  (3301 - 2644.53) |
| 85-89 | 544.9  (577.63 - 487.09) | 475.37  (518.96 - 408.57) | 484.39  (517.15 - 431.67) | 386.48  (422.09 - 332.64) | 5104.74  (5463.42 - 4569.5) | 4093.14  (4493.56 - 3502.86) |
| 90-94 | 538.98  (584.31 - 472.14) | 487.88  (533.83 - 414.55) | 703.17  (762.42 - 619.12) | 622.13  (679.45 - 533.32) | 6254.91  (6773.34 - 5521.38) | 5539.02  (6047.28 - 4735.32) |
| 95+ | 518.13  (571.69 - 416.1) | 500.25  (561.7 - 383.17) | 726.29  (799.33 - 585.85) | 684.23  (766.45 - 525.93) | 6089.62  (6706.04 - 4940.86) | 5690.85  (6365.24 - 4394.09) |

Abbreviations: UI, uncertainty interval; DALYs, disability adjusted life years.

Supplementary table 3 ASR for incidence, mortality, and DALYs of prostate cancer in 204 countries and territories in 2021 and the trends from 1990-2021.

| Location | Incidence | | Mortality | | DALYs | |
| --- | --- | --- | --- | --- | --- | --- |
|  | 2021  (95% UI) | EAPC  (95% CI) | 2021  (95% UI) | EAPC  (95% CI) | 2021  (95% UI) | EAPC  (95% CI) |
| American Samoa | 205.65  (133.94-303.02) | 0.909  (0.722-1.096) | 156.62  (104.28-224.98) | 0.541  (0.343,0.740) | 432.81  (252.46-684.12) | 0.550  (0.520,0.580) |
| Antigua and Barbuda | 506.14  (370.72-668.21) | 0.648  (0.239-1.059) | 228.15  (186.75-274.24) | 0.289  (-0.131,0.712) | 2542.76  (1693.42-3664.44) | 0.527  (0.324,0.731) |
| Arab Republic of Egypt | 51.94  (29.92-80.86) | 4.073  (3.809-4.339) | 23.63  (14.65-35.74) | 2.585  (2.240,2.932) | 1447.96  (820.7-2272.14) | 0.696  (0.620,0.772) |
| Argentine Republic | 112.7  (85.4-145.18) | 0.471  (0.028-0.916) | 64.85  (51.73-78.02) | -0.578  (-0.938,-0.216) | 3754.66  (3026.74-4561.08) | -0.067  (-0.453,0.321) |
| Australia | 292.32  (211.47-393.27) | -0.820  (-1.579--0.055) | 58.45  (46.28-71.56) | -2.897  (-3.280,-2.513) | 418.14  (253.22-629.28) | 2.478  (2.180,2.778) |
| Barbados | 358.77  (241.47-505.21) | 0.740  (0.561-0.920) | 154.83  (111.71-200.49) | -0.046  (-0.193,0.101) | 1114.34  (891.77-1342.27) | -0.637  (-1.007,-0.265) |
| Belize | 189.7  (143.87-246.1) | 1.614  (0.877-2.356) | 101.98  (81.38-123.82) | 0.944  (0.085,1.811) | 1048.6  (829.13-1308.08) | -2.802  (-3.263,-2.339) |
| Bermuda | 584.98  (405.13-828.26) | 1.385  (1.078-1.693) | 132.42  (101.79-169.93) | -0.755  (-0.915,-0.594) | 282.74  (132.8-534.94) | -0.660  (-0.806,-0.515) |
| Bolivarian Republic of Venezuela | 300.95  (199.89-433.75) | 1.924  (1.424-2.427) | 96.51  (70.68-126.75) | 0.529  (0.132,0.926) | 2583.74  (1849.8-3393.89) | -0.053  (-0.189,0.082) |
| Bosnia and Herzegovina | 98.18  (59.2-145.75) | 2.877  (2.580-3.173) | 47.85  (30-67.83) | 1.050  (0.891,1.209) | 1785.16  (1427.08-2178.01) | 0.926  (0.094,1.764) |
| Brunei Darussalam | 63.78  (39.15-97.12) | 1.600  (1.405-1.795) | 44.59  (27.44-66.98) | 0.982  (0.663,1.301) | 1448.93  (748.89-2347.26) | 1.677  (1.570,1.784) |
| Burkina Faso | 83.65  (43.46-136.91) | 2.172  (2.056-2.289) | 81.73  (43.14-132.61) | 1.946  (1.838,2.055) | 2328.12  (1774.01-3034.76) | -0.683  (-0.843,-0.523) |
| Canada | 158.1  (116.18-212.25) | -2.217  (-2.573--1.859) | 47.76  (38.53-57.98) | -3.147  (-3.373,-2.921) | 275.36  (137.39-522.15) | 0.004  (-0.068,0.076) |
| Central African Republic | 72.91  (39.93-119.3) | 0.447  (0.419-0.476) | 78.14  (42.38-128.61) | 0.373  (0.341,0.405) | 1770.12  (1274.37-2373.14) | 0.458  (0.073,0.844) |
| Commonwealth of Dominica | 309.81  (183.04-485.34) | 0.354  (-0.026-0.736) | 192.32  (119.62-293.75) | 0.122  (-0.194,0.440) | 860.48  (543.33-1221.66) | 1.195  (1.033,1.357) |
| Commonwealth of the Bahamas | 405.21  (300.6-542.53) | 0.972  (0.668-1.276) | 204.3  (162.21-257.02) | 0.386  (0.111,0.661) | 2275.33  (1402.89-3482.68) | 0.265  (-0.033,0.564) |
| Cook Islands | 375.53  (241.09-554.38) | 1.153  (1.019-1.286) | 185.13  (123.62-261.62) | -0.402  (-0.471,-0.332) | 678.33  (427.32-1010.63) | 0.527  (0.303,0.752) |
| Czech Republic | 183.7  (135.24-243.26) | 1.370  (0.819-1.924) | 58.6  (47.57-70.48) | -0.853  (-1.207,-0.497) | 1383.76  (722.8-2275.12) | 1.865  (1.768,1.963) |
| Democratic People's Republic of Korea | 25.23  (15.31-39.41) | 1.488  (1.364-1.612) | 15.94  (9.81-24.35) | 0.420  (0.313,0.527) | 1207.98  (573.71-2110.54) | -0.568  (-0.669,-0.467) |
| Democratic Republic of Sao Tome and Principe | 82.7  (48.62-131.23) | 1.738  (1.544-1.932) | 70.36  (41.01-111.46) | 1.108  (0.896,1.319) | 2679.56  (1478.12-4710) | 2.241  (1.777,2.707) |
| Democratic Republic of the Congo | 80.21  (44.83-131.35) | 0.887  (0.685-1.089) | 79.42  (44.72-131.7) | 0.639  (0.473,0.805) | 1675.23  (766.51-2792.38) | 1.722  (1.612,1.832) |
| Democratic Republic of Timor-Leste | 32.57  (18.92-52.71) | 1.657  (1.573-1.742) | 28.53  (16.77-45.84) | 1.058  (1.006,1.110) | 793.26  (639.54-974.08) | -3.275  (-3.552,-2.998) |
| Democratic Socialist Republic of Sri Lanka | 38.23  (20.91-62.95) | 1.981  (1.847-2.116) | 18.3  (10.71-28.79) | 0.055  (-0.080,0.189) | 1345.1  (720.09-2220.49) | 0.317  (0.293,0.341) |
| Dominican Republic | 156.29  (84.95-277.42) | 1.072  (0.588-1.558) | 94.27  (53.45-159.17) | 0.250  (-0.158,0.660) | 1330.89  (671.47-2201.33) | 2.251  (2.067,2.435) |
| Eastern Republic of Uruguay | 196.28  (148.46-256.83) | 0.757  (0.362-1.153) | 106.29  (86.12-128.93) | -0.332  (-0.600,-0.064) | 3178.12  (1966.8-4875.97) | 0.060  (-0.272,0.392) |
| Federal Democratic Republic of Ethiopia | 28.72  (14.59-46.23) | 0.533  (0.366-0.701) | 26.24  (13.52-42.06) | 0.019  (-0.104,0.141) | 3524.66  (2764.89-4481.26) | 0.230  (0.012,0.449) |
| Federal Democratic Republic of Nepal | 18.34  (9.45-34.2) | 0.945  (0.771-1.120) | 15.63  (8.19-28.82) | 0.264  (0.098,0.430) | 1419.82  (731.83-2393.99) | 0.151  (0.088,0.214) |
| Federal Republic of Germany | 249.81  (185.44-327.98) | 0.517  (0.101-0.935) | 56.75  (45.92-68.21) | -1.834  (-2.108,-1.558) | 1569.48  (862.65-2471.13) | 0.110  (0.010,0.211) |
| Federal Republic of Nigeria | 155.46  (64.09-234.75) | 1.098  (1.005-1.192) | 143.38  (62.66-211.24) | 0.650  (0.579,0.721) | 3052.27  (2038.65-4342.75) | -0.311  (-0.381,-0.242) |
| Federal Republic of Somalia | 60.63  (27.2-114.37) | 0.013  (-0.044-0.070) | 60.61  (27.09-114.43) | -0.088  (-0.149,-0.026) | 1051.07  (853.61-1269.06) | -0.660  (-0.992,-0.327) |
| Federated States of Micronesia | 106.06  (66.23-160.83) | 1.159  (1.104-1.213) | 94.1  (59.44-140.23) | 0.664  (0.618,0.710) | 2304.41  (1380.47-3573.12) | 0.225  (0.124,0.327) |
| Federative Republic of Brazil | 122.7  (108.43-135.66) | 0.920  (0.545-1.295) | 64.24  (56.87-69.99) | -0.076  (-0.418,0.268) | 274.68  (170.44-416.62) | 0.493  (0.389,0.598) |
| French Republic | 268.96  (192.23-363.88) | 0.734  (0.476-0.993) | 54.23  (42.64-66.93) | -2.689  (-2.900,-2.477) | 1323.18  (754.74-2159.76) | 0.581  (0.417,0.746) |
| Gabonese Republic | 120.52  (62.6-200.04) | 0.975  (0.935-1.015) | 106.08  (55.42-173.67) | 0.437  (0.413,0.461) | 478.99  (279.8-767.84) | 1.004  (0.948,1.061) |
| Georgia | 136.1  (107.31-169.91) | 4.353  (3.594-5.118) | 80.15  (67.62-94.57) | 4.102  (3.204,5.009) | 322.67  (184.4-514.35) | 0.151  (0.023,0.279) |
| Grand Duchy of Luxembourg | 172.28  (126.91-230.45) | -0.137  (-0.410-0.136) | 50.23  (41.28-59.82) | -2.217  (-2.343,-2.092) | 1580.71  (737.32-2747.53) | 0.239  (0.160,0.318) |
| Greenland | 62  (38.38-94.6) | 0.052  (-0.035-0.138) | 37.61  (23.83-56.21) | -1.056  (-1.133,-0.980) | 1579.81  (885.34-2709.3) | 0.391  (0.016,0.769) |
| Grenada | 490.47  (361.59-647.6) | 2.081  (0.612-3.571) | 280.29  (220.3-349.97) | 2.114  (0.506,3.747) | 1756.54  (1419.53-2138.49) | -0.410  (-0.716,-0.103) |
| Guam | 73.9  (48.6-110.43) | 0.541  (0.226-0.857) | 37.36  (25.55-54.66) | -0.412  (-0.713,-0.111) | 1591.31  (813.48-2612.93) | 0.747  (0.658,0.837) |
| Hashemite Kingdom of Jordan | 73.41  (40.4-119.49) | 2.796  (2.512-3.081) | 22.49  (13.23-34.71) | 0.069  (-0.078,0.217) | 1483.21  (688.81-2480.84) | 0.224  (0.097,0.350) |
| Hellenic Republic | 168.71  (126.87-219.87) | -0.328  (-0.685-0.030) | 53.21  (44.59-61.8) | -1.392  (-1.701,-1.081) | 2420.02  (1324.84-4103.19) | 0.971  (0.734,1.207) |
| Hungary | 137.88  (102.71-178.51) | 0.687  (0.382-0.994) | 55.28  (44.88-65.53) | -1.174  (-1.419,-0.929) | 461.15  (235.22-738.62) | -0.173  (-0.302,-0.043) |
| Independent State of Papua New Guinea | 66.64  (33.92-110.63) | 1.015  (0.980-1.050) | 62.62  (31.91-103.44) | 0.883  (0.842,0.924) | 1042.62  (843.44-1259.46) | -1.493  (-1.720,-1.267) |
| Independent State of Samoa | 58.22  (31.07-92.56) | 0.364  (0.336-0.393) | 44.04  (24.17-68.94) | -0.199  (-0.260,-0.137) | 1542.65  (969.9-2330.63) | 0.643  (0.600,0.685) |
| Ireland | 190.93  (134.15-261.19) | 0.258  (-0.248-0.766) | 51.48  (39.94-63.75) | -2.147  (-2.472,-1.821) | 1114.07  (1003.44-1210.94) | -0.148  (-0.492,0.197) |
| Islamic Republic of Afghanistan | 31.99  (18.43-50.94) | 1.249  (1.136-1.362) | 25.41  (14.81-40.28) | 0.581  (0.548,0.613) | 969.17  (754.7-1213.04) | -2.241  (-2.427,-2.054) |
| Islamic Republic of Iran | 89.38  (55.05-120.08) | 2.905  (2.720-3.091) | 31.79  (20.88-39.21) | 0.847  (0.678,1.016) | 1749.18  (906.99-2888.38) | 0.382  (0.363,0.402) |
| Islamic Republic of Mauritania | 109.45  (53.77-183.13) | 2.463  (2.364-2.563) | 91.37  (43.82-150.64) | 1.803  (1.707,1.899) | 367.44  (218.03-578.68) | 0.743  (0.642,0.845) |
| Islamic Republic of Pakistan | 37.12  (23.77-55.47) | 1.156  (0.997-1.314) | 33.73  (21.7-50.61) | 0.762  (0.567,0.958) | 1538.58  (1298.01-1818.5) | 3.982  (3.183,4.788) |
| Jamaica | 377.42  (251.41-546.28) | 2.207  (1.413-3.007) | 169.11  (120.93-225.55) | 1.595  (0.899,2.296) | 1916.95  (1194.18-2871.08) | 0.127  (-0.029,0.283) |
| Japan | 88.71  (68.73-109.49) | 2.277  (1.731-2.826) | 22.95  (19.98-24.63) | -0.132  (-0.431,0.167) | 839.14  (685.65-1009.84) | -2.191  (-2.309,-2.073) |
| Kingdom of Bahrain | 184.42  (102.33-306.11) | 1.912  (1.750-2.074) | 58.77  (36.54-91.03) | -0.737  (-1.019,-0.455) | 643.14  (411.07-966.25) | -1.064  (-1.142,-0.986) |
| Kingdom of Belgium | 185.94  (134.59-249.37) | -0.788  (-1.186--0.389) | 53.53  (41.88-65.42) | -2.748  (-2.911,-2.584) | 4596.17  (3593.57-5754.12) | 1.517  (0.083,2.971) |
| Kingdom of Bhutan | 20.04  (9.95-38.77) | 0.930  (0.826-1.033) | 16.38  (8.36-30.81) | 0.103  (0.036,0.169) | 707.34  (488.99-1031.26) | -0.114  (-0.375,0.147) |
| Kingdom of Cambodia | 54.46  (31.93-84.09) | 1.969  (1.859-2.079) | 45.34  (26.76-69.58) | 1.238  (1.149,1.327) | 987.58  (582.16-1537.81) | 1.003  (0.927,1.079) |
| Kingdom of Denmark | 223.29  (166.12-293.81) | 1.717  (1.129-2.308) | 88.67  (70.84-106.41) | -0.715  (-0.901,-0.528) | 1692.78  (846.11-2817.17) | 1.808  (1.687,1.930) |
| Kingdom of Eswatini | 147.91  (81.52-249.52) | 1.198  (1.023-1.373) | 127.75  (70.05-214.26) | 0.896  (0.657,1.135) | 399.91  (233.81-624.38) | 0.181  (0.066,0.296) |
| Kingdom of Lesotho | 141.58  (83.22-225.29) | 1.307  (1.166-1.447) | 132.07  (78.49-208.97) | 1.187  (1.002,1.372) | 900.72  (756.42-1054.39) | -1.337  (-1.614,-1.059) |
| Kingdom of Morocco | 36.76  (17.97-61.98) | 2.384  (2.138-2.630) | 18.17  (9.05-30.4) | 0.728  (0.574,0.882) | 1021.73  (825.38-1216.22) | -0.899  (-1.133,-0.665) |
| Kingdom of Norway | 244  (186.56-311.64) | 0.360  (-0.182-0.905) | 74.39  (63.79-83.15) | -1.541  (-1.749,-1.331) | 1035.66  (528.18-1715.71) | 0.830  (0.786,0.874) |
| Kingdom of Saudi Arabia | 60.99  (30.31-125.51) | 2.707  (2.496-2.919) | 14.04  (7.57-27.45) | -0.656  (-0.977,-0.334) | 743.71  (396.08-1172.64) | -0.175  (-0.237,-0.113) |
| Kingdom of Spain | 153.16  (109.8-206.24) | 0.335  (-0.107-0.778) | 43.37  (33.89-53.15) | -2.152  (-2.307,-1.997) | 287.43  (223.07-399.65) | 0.220  (0.053,0.387) |
| Kingdom of Sweden | 230.17  (169.56-304.52) | -0.297  (-0.789-0.197) | 71.87  (57.08-86.97) | -1.520  (-1.806,-1.233) | 869.8  (671.93-1083.38) | -2.092  (-2.445,-1.738) |
| Kingdom of Thailand | 69.47  (34.87-112.5) | 1.838  (1.683-1.992) | 30.33  (16.19-46.98) | -0.276  (-0.413,-0.139) | 559.28  (363.9-687.54) | 0.946  (0.778,1.114) |
| Kingdom of the Netherlands | 207.85  (154.07-275.5) | 0.197  (-0.162-0.556) | 61.46  (49.89-73.99) | -2.058  (-2.244,-1.871) | 2990.84  (2103.81-4051.45) | 1.604  (0.868,2.345) |
| Kingdom of Tonga | 153.43  (97.06-228.65) | 0.748  (0.638-0.859) | 116.41  (76.03-170.15) | 0.268  (0.158,0.378) | 390.1  (344.92-427.67) | -0.027  (-0.320,0.266) |
| Kyrgyz Republic | 34.96  (25.52-46.42) | 0.441  (-0.051-0.936) | 22.54  (17.16-28.53) | -0.340  (-0.823,0.145) | 910.7  (578.9-1229.01) | 1.911  (1.672,2.150) |
| Lao People's Democratic Republic | 37.78  (22.18-59.76) | 1.204  (1.164-1.244) | 33.72  (19.78-53.39) | 0.631  (0.595,0.668) | 987.6  (599.97-1543.62) | -0.602  (-0.841,-0.362) |
| Lebanese Republic | 189.75  (109.62-302) | 3.860  (3.513-4.209) | 54.82  (33.83-82.45) | 1.227  (0.918,1.537) | 917.8  (723.54-1135.76) | -2.503  (-2.739,-2.266) |
| Malaysia | 55.15  (32.91-85.55) | 1.613  (1.432-1.794) | 30.84  (18.71-46.46) | 0.261  (0.020,0.501) | 762.03  (445.77-1173.3) | 1.106  (1.028,1.184) |
| Mongolia | 17.84  (11.1-26.95) | 2.477  (2.325-2.629) | 13.03  (8.14-19.5) | 1.360  (1.281,1.439) | 1423.37  (1143.3-1719.77) | -0.881  (-1.130,-0.631) |
| Montenegro | 205.33  (133.3-307.86) | 1.837  (1.447-2.228) | 91.1  (63.07-129.81) | 0.793  (0.350,1.238) | 1187.03  (1019.22-1335.83) | -1.689  (-1.955,-1.422) |
| New Zealand | 292.22  (216.83-390.33) | -0.971  (-1.217--0.725) | 62.09  (49.12-75.65) | -1.894  (-2.132,-1.655) | 740.07  (580.83-910.17) | -2.049  (-2.199,-1.899) |
| North Macedonia | 96.05  (58.45-144.54) | 2.220  (1.832-2.610) | 51.51  (31.8-75.78) | 0.828  (0.410,1.248) | 1154.21  (910.65-1412.59) | -1.624  (-1.923,-1.324) |
| Northern Mariana Islands | 134.13  (85.85-199.31) | 1.580  (1.321-1.840) | 82.94  (54.44-121.12) | 1.154  (0.900,1.408) | 555.27  (290.81-870.41) | 0.005  (-0.113,0.124) |
| Palestine | 134.41  (82.34-215.06) | 1.428  (1.260-1.597) | 58.67  (38.04-89.75) | -0.222  (-0.516,0.072) | 1074.86  (879.07-1296.35) | -1.718  (-1.919,-1.516) |
| People's Democratic Republic of Algeria | 19.87  (10.75-32.97) | 1.872  (1.779-1.966) | 8.61  (4.6-13.95) | 0.043  (-0.027,0.114) | 1880.16  (1231.3-2763.66) | 0.272  (0.165,0.378) |
| People's Republic of Bangladesh | 21.29  (10.06-40.66) | 0.412  (0.270-0.555) | 16.53  (7.99-30.93) | -0.510  (-0.701,-0.318) | 423.27  (319.49-537.94) | -0.525  (-0.886,-0.162) |
| People's Republic of China | 27.63  (18.78-37.76) | 1.832  (1.702-1.961) | 14.56  (10.14-20.04) | -0.333  (-0.564,-0.101) | 566.56  (331.5-893.65) | 0.488  (0.454,0.522) |
| Plurinational State of Bolivia | 121.6  (69.05-203.02) | 1.011  (0.962-1.060) | 90.86  (53.41-148.27) | 0.193  (0.144,0.241) | 933.7  (574.68-1403.19) | 1.282  (0.989,1.576) |
| Portuguese Republic | 214.05  (152.45-291.61) | 0.677  (0.344-1.010) | 57.04  (45-69.91) | -2.109  (-2.360,-1.858) | 2506.45  (1486.42-3995.87) | 1.280  (1.122,1.437) |
| Principality of Andorra | 242.4  (130.32-408.95) | 1.065  (0.751-1.381) | 63.01  (37.79-98.44) | -0.624  (-0.846,-0.402) | 1312.85  (637.57-2242.69) | 1.699  (1.593,1.805) |
| Principality of Monaco | 264.14  (155.97-419.59) | 1.170  (1.013-1.326) | 72.3  (45.9-110.54) | -0.230  (-0.278,-0.181) | 726.17  (393.91-1178) | 0.548  (0.459,0.638) |
| Puerto Rico | 231.84  (160.87-324.74) | 0.099  (-0.175-0.373) | 56.82  (43.34-72.64) | -2.213  (-2.446,-1.980) | 1002.79  (490.47-1719.42) | -0.544  (-0.677,-0.409) |
| Republic of Albania | 83.02  (50.55-131.65) | 1.304  (1.152-1.455) | 46.87  (29.8-71.52) | -0.478  (-0.626,-0.331) | 1190.79  (739.2-1806.56) | 1.124  (0.975,1.274) |
| Republic of Angola | 89.15  (50.07-141.06) | 1.159  (1.051-1.266) | 86.64  (48.83-136.44) | 0.802  (0.731,0.873) | 546.75  (334.83-818.98) | 0.249  (0.090,0.407) |
| Republic of Armenia | 90.65  (70.8-114.34) | 2.858  (2.603-3.115) | 46.16  (38.21-54.39) | 1.694  (1.424,1.964) | 627.97  (386.01-980.41) | 0.669  (0.567,0.771) |
| Republic of Austria | 220.08  (158.68-299.93) | -0.037  (-0.435-0.362) | 51.18  (40.37-61.76) | -1.926  (-2.066,-1.786) | 1544.06  (742.96-2564.09) | 1.710  (1.612,1.808) |
| Republic of Azerbaijan | 43.63  (29.08-63.52) | 0.877  (0.638-1.116) | 26.86  (18.13-38.97) | -0.242  (-0.461,-0.022) | 900.91  (747.54-1068.92) | 0.301  (0.004,0.599) |
| Republic of Belarus | 230.95  (159.46-322.96) | 3.368  (3.043-3.694) | 63.08  (48.19-81.57) | 1.245  (0.979,1.512) | 1248.27  (794.61-1893.67) | -0.144  (-0.193,-0.096) |
| Republic of Benin | 90.4  (46.78-145.7) | 2.068  (1.958-2.178) | 86.32  (45.13-138.56) | 1.773  (1.655,1.891) | 251.07  (158.99-372.46) | 1.440  (1.356,1.524) |
| Republic of Botswana | 150.37  (91.08-230.51) | 0.784  (0.557-1.012) | 129.26  (80.48-196.49) | 0.331  (0.045,0.618) | 1560.98  (1071.79-2234.03) | 0.795  (0.473,1.118) |
| Republic of Bulgaria | 135.66  (105.7-172.54) | 2.598  (2.441-2.755) | 69.31  (58.56-80.21) | 1.283  (1.108,1.457) | 317.81  (156.86-526.06) | 0.916  (0.754,1.079) |
| Republic of Burundi | 68.53  (32.24-119.22) | -0.189  (-0.282--0.097) | 66.56  (31.94-115.09) | -0.402  (-0.492,-0.313) | 592.03  (347.06-953.07) | 1.047  (0.964,1.130) |
| Republic of Cabo Verde | 222.35  (120.28-390.99) | 3.272  (2.865-3.681) | 159.56  (86.32-281.9) | 2.242  (1.784,2.701) | 2134.86  (1256-3255) | 2.262  (2.033,2.492) |
| Republic of Cameroon | 105.8  (49.4-174.77) | 2.084  (2.001-2.167) | 97.66  (45.18-161.17) | 1.751  (1.648,1.854) | 1608.86  (923.8-2686.07) | 0.646  (0.576,0.716) |
| Republic of Chad | 76.53  (39.28-126.71) | 2.452  (2.288-2.615) | 77.14  (39.35-127.56) | 2.276  (2.100,2.453) | 265.56  (137.91-489.95) | 0.201  (0.030,0.372) |
| Republic of Chile | 156.82  (116.91-203.52) | 1.761  (1.387-2.136) | 70.09  (56.68-83.89) | -0.089  (-0.469,0.292) | 1107.96  (883.75-1366.74) | -1.955  (-2.163,-1.747) |
| Republic of Colombia | 225.94  (156.25-315.48) | 1.047  (0.687-1.409) | 53.08  (41.21-67.24) | -1.429  (-1.706,-1.150) | 1203.34  (567.48-2150.42) | 1.884  (1.755,2.013) |
| Republic of Costa Rica | 311.56  (219.88-427.74) | 2.376  (1.987-2.766) | 65.78  (52.26-81.2) | 0.629  (0.163,1.097) | 2395.6  (1010.6-3613.35) | 0.614  (0.544,0.683) |
| Republic of Croatia | 199.66  (146.78-264.22) | 1.387  (1.126-1.649) | 73.19  (58.95-89.11) | -0.534  (-0.750,-0.317) | 1537.48  (950.94-2342.4) | 0.568  (0.528,0.609) |
| Republic of Cuba | 375.25  (265.08-514.08) | 2.071  (1.951-2.192) | 117.59  (92.32-147.12) | 0.734  (0.640,0.827) | 892.32  (558.21-1302.33) | 0.776  (0.455,1.098) |
| Republic of Cyprus | 246.23  (146.18-391.66) | 1.410  (1.068-1.754) | 68.59  (40.44-103.01) | -1.849  (-2.245,-1.451) | 1345.52  (886.85-1957.2) | 1.199  (0.978,1.420) |
| Republic of C涔坱e d'Ivoire | 146.71  (88.67-225.76) | 0.648  (0.585-0.711) | 132.82  (81.11-202.81) | 0.252  (0.155,0.350) | 241.46  (141.56-394.28) | 0.403  (0.193,0.614) |
| Republic of Djibouti | 98.16  (47.19-167.48) | 0.700  (0.660-0.739) | 86.6  (40.71-147.86) | 0.318  (0.247,0.388) | 585.88  (374.09-875.45) | 0.727  (0.533,0.921) |
| Republic of Ecuador | 117.02  (81.89-162.39) | 1.209  (0.775-1.645) | 70.78  (53.7-91.81) | 0.174  (-0.231,0.581) | 1466.29  (878.87-2311.44) | -0.547  (-0.593,-0.501) |
| Republic of El Salvador | 216.7  (135.28-336.24) | 2.877  (2.422-3.335) | 64.8  (44.82-93.4) | 0.469  (0.204,0.734) | 946.91  (612.45-1453.1) | -0.145  (-0.397,0.107) |
| Republic of Equatorial Guinea | 114.07  (58.87-188.78) | 1.860  (1.768-1.953) | 95.78  (49.45-156) | 0.904  (0.836,0.971) | 137.91  (76.68-220.68) | 0.014  (-0.045,0.072) |
| Republic of Estonia | 420.04  (297.62-581.28) | 4.142  (3.403-4.886) | 101.68  (79.71-126.11) | 1.569  (0.847,2.296) | 227.06  (159.45-314.58) | -0.281  (-0.475,-0.087) |
| Republic of Fiji | 82.33  (25.91-147.89) | 0.619  (0.476-0.763) | 73.15  (22.92-131.03) | 0.320  (0.144,0.495) | 1500.21  (873.43-2470.29) | 0.112  (0.066,0.159) |
| Republic of Finland | 289.06  (207.79-387.03) | 0.866  (0.320-1.415) | 57.03  (44.76-69.4) | -1.817  (-2.011,-1.624) | 992.56  (781.75-1224.83) | -1.808  (-2.039,-1.577) |
| Republic of Ghana | 125.85  (77.91-192.88) | 0.519  (0.340-0.699) | 110.27  (68.82-165.32) | 0.073  (-0.093,0.239) | 1097.86  (645.05-1730.31) | -0.482  (-0.704,-0.260) |
| Republic of Guatemala | 138.39  (102.73-181.15) | 2.232  (1.401-3.069) | 68.9  (55.99-83.61) | 0.623  (-0.152,1.404) | 1062.2  (805.56-1370.32) | -1.784  (-2.001,-1.565) |
| Republic of Guinea | 58.12  (34.6-90.8) | 1.241  (1.173-1.310) | 56.73  (33.68-87.18) | 0.996  (0.925,1.067) | 1058.37  (593.03-1734.36) | -0.436  (-0.840,-0.031) |
| Republic of Guinea-Bissau | 97.02  (48.64-160.45) | 2.120  (2.006-2.233) | 97.12  (48.94-159.01) | 1.912  (1.789,2.036) | 756.57  (480.86-1158.12) | -0.375  (-0.511,-0.239) |
| Republic of Guyana | 227.39  (157.05-315.99) | 1.018  (0.737-1.299) | 157.17  (113.9-209.68) | 0.584  (0.278,0.891) | 886.02  (735.39-1041.4) | 1.556  (1.320,1.793) |
| Republic of Haiti | 177.25  (105.51-276.83) | 0.708  (0.663-0.752) | 160.73  (94.45-247.41) | 0.436  (0.388,0.484) | 909.72  (719.11-1112.24) | -1.742  (-1.880,-1.604) |
| Republic of Honduras | 108.62  (56.76-203.23) | 2.086  (2.010-2.163) | 58.15  (31.59-105.7) | 1.046  (0.955,1.136) | 530.54  (361.8-755.49) | -0.131  (-0.309,0.048) |
| Republic of Iceland | 260.15  (187.32-354.8) | -0.134  (-0.475-0.207) | 68.27  (53.29-85.13) | -1.166  (-1.354,-0.978) | 1287.42  (968.57-1694.99) | 1.383  (1.088,1.678) |
| Republic of India | 21.49  (16.48-29.66) | 1.214  (1.032-1.395) | 16.65  (12.87-23.19) | 0.432  (0.258,0.606) | 1219.39  (1024.81-1424.31) | 1.248  (1.071,1.426) |
| Republic of Indonesia | 50.23  (30.27-71.99) | 2.162  (2.113-2.210) | 39.29  (23.88-56.38) | 1.568  (1.480,1.656) | 1168.04  (948.1-1397.64) | -0.239  (-0.550,0.074) |
| Republic of Iraq | 55.6  (31.89-90.78) | 3.116  (2.783-3.451) | 21.67  (13.17-34.07) | 1.016  (0.811,1.222) | 1005.55  (774.43-1285.09) | -1.248  (-1.527,-0.969) |
| Republic of Italy | 178.8  (136.25-227.33) | 0.217  (-0.259-0.695) | 42.57  (36.73-46.96) | -1.311  (-1.557,-1.065) | 1236.96  (981.14-1541.67) | 0.878  (0.479,1.278) |
| Republic of Kazakhstan | 53.39  (42.09-66.58) | 1.770  (1.362-2.179) | 30.02  (25.28-34.98) | 0.571  (0.270,0.874) | 1259.5  (1010.96-1538.62) | -0.301  (-0.524,-0.079) |
| Republic of Kenya | 57.87  (37.01-77.66) | 2.230  (2.040-2.421) | 49.74  (31.4-67.15) | 2.022  (1.780,2.265) | 2102.48  (1637.71-2648.19) | 0.949  (0.864,1.035) |
| Republic of Kiribati | 41.64  (23.9-66.21) | 0.696  (0.671-0.721) | 41.48  (23.33-66.18) | 0.506  (0.469,0.543) | 1140.69  (709.44-1722.56) | -1.369  (-1.638,-1.098) |
| Republic of Korea | 76.91  (41.56-116.79) | 4.352  (3.741-4.967) | 22.91  (12.59-33.73) | 0.575  (0.209,0.942) | 1109.88  (820.61-1467.56) | 0.088  (-0.331,0.509) |
| Republic of Latvia | 270.36  (193.67-365.26) | 3.474  (3.193-3.755) | 100.22  (78.14-123.56) | 2.354  (2.060,2.648) | 1187.14  (807.35-1724.54) | 0.661  (0.401,0.921) |
| Republic of Liberia | 84.51  (41.56-143.91) | 2.232  (2.113-2.351) | 77.5  (38.11-131.59) | 1.709  (1.603,1.815) | 1942.39  (1505.17-2440.42) | 1.666  (0.956,2.380) |
| Republic of Lithuania | 403.68  (285.78-550.43) | 3.513  (2.967-4.061) | 87.51  (69.4-107.86) | 2.186  (1.669,2.707) | 1134.25  (357.43-2055.9) | 0.351  (0.230,0.471) |
| Republic of Madagascar | 58.89  (28.54-100.88) | -0.197  (-0.332--0.061) | 54.74  (26.76-92.7) | -0.431  (-0.562,-0.300) | 1057.03  (834.51-1297.54) | -1.605  (-1.829,-1.380) |
| Republic of Malawi | 70.96  (43.74-108.5) | 1.540  (1.414-1.666) | 63.95  (39.68-96.83) | 1.121  (0.980,1.262) | 1139.27  (914.25-1401.98) | 0.803  (0.072,1.539) |
| Republic of Maldives | 35.39  (17.97-58.46) | 1.263  (1.085-1.441) | 19.63  (9.85-31.69) | -0.623  (-0.743,-0.502) | 2786.33  (1997.56-3761.81) | 0.606  (0.334,0.879) |
| Republic of Mali | 38.15  (23.37-59.5) | 1.000  (0.904-1.096) | 36.44  (22.46-56.1) | 0.679  (0.580,0.779) | 2686.92  (1574.48-4141.12) | 0.377  (0.340,0.415) |
| Republic of Malta | 134.58  (95.18-186.07) | 0.113  (-0.162-0.388) | 32.25  (24.96-40.85) | -2.058  (-2.250,-1.865) | 986.76  (536.16-1797.72) | 1.149  (1.054,1.244) |
| Republic of Mauritius | 92.28  (71.83-116.28) | 1.167  (0.819-1.517) | 50.84  (41.97-60.21) | 0.243  (-0.026,0.513) | 1164.35  (913.87-1455.82) | -1.325  (-1.516,-1.133) |
| Republic of Moldova | 130.17  (97.91-169.31) | 3.875  (3.164-4.590) | 42.31  (35.07-50.42) | 1.899  (1.284,2.518) | 659.97  (402.71-935.97) | 1.414  (1.337,1.491) |
| Republic of Mozambique | 34.75  (20.37-55.93) | 1.226  (1.159-1.293) | 34.97  (20.51-55.7) | 1.019  (0.939,1.099) | 378.15  (229.05-593.87) | 1.033  (0.841,1.226) |
| Republic of Namibia | 144.42  (84.44-221.56) | 2.952  (2.721-3.184) | 114.17  (68.78-171.13) | 2.279  (2.040,2.518) | 733.73  (633.93-824.55) | -1.405  (-1.632,-1.178) |
| Republic of Nauru | 111.87  (63.82-188.09) | 0.999  (0.961-1.038) | 97.14  (57.39-156.93) | 0.661  (0.598,0.724) | 568.84  (478.71-666.47) | 0.385  (0.109,0.663) |
| Republic of Nicaragua | 137.85  (84.46-212.31) | 1.901  (1.604-2.199) | 45.41  (28.98-66.53) | -0.216  (-0.547,0.116) | 692.16  (399.31-1089.65) | 0.444  (0.400,0.487) |
| Republic of Niue | 131.92  (80.28-204.89) | 1.284  (1.227-1.341) | 95.45  (59.58-144.25) | 0.586  (0.546,0.626) | 369.62  (211.33-534.48) | 0.728  (0.390,1.066) |
| Republic of Palau | 124.86  (74.04-198.77) | 0.021  (-0.031-0.072) | 85.91  (51.66-134.75) | -0.622  (-0.693,-0.552) | 1849.23  (1426.85-2292.09) | 2.258  (1.936,2.581) |
| Republic of Panama | 265.27  (174.86-381.33) | 1.738  (1.161-2.319) | 61.62  (45.02-79.32) | 0.010  (-0.548,0.571) | 1768.39  (1382.36-2198.14) | 2.351  (1.794,2.911) |
| Republic of Paraguay | 136.28  (73.46-222.6) | 2.332  (1.974-2.691) | 75.25  (41.9-120.77) | 1.597  (1.236,1.960) | 314.12  (162.18-503.61) | -0.833  (-0.961,-0.705) |
| Republic of Peru | 132.66  (79.25-213.54) | 1.769  (1.626-1.913) | 60.25  (38.1-94.42) | -0.273  (-0.404,-0.141) | 581.73  (449.31-742.96) | -1.905  (-2.099,-1.710) |
| Republic of Poland | 144.99  (117.82-177.03) | 2.675  (2.311-3.039) | 77.53  (67.86-85.93) | 0.992  (0.612,1.373) | 934.12  (770.01-1118.54) | 2.390  (1.806,2.978) |
| Republic of Rwanda | 92.21  (45.78-155.54) | 0.234  (0.111-0.358) | 83.14  (41.78-138.04) | -0.283  (-0.404,-0.162) | 826.12  (533-1213.9) | 0.133  (-0.174,0.442) |
| Republic of San Marino | 141.46  (73.96-246.88) | -0.176  (-0.646-0.296) | 35.39  (20.62-57.2) | -1.433  (-1.924,-0.939) | 1136.27  (812.17-1491.43) | 0.161  (-0.361,0.687) |
| Republic of Senegal | 106.49  (54.61-174.53) | 2.524  (2.399-2.649) | 98.05  (49.5-159.08) | 2.197  (2.076,2.318) | 1287.55  (714.55-2090.2) | 1.510  (1.148,1.874) |
| Republic of Serbia | 122.23  (75.71-182.58) | 1.803  (1.575-2.031) | 56.67  (35.49-82.27) | -0.166  (-0.319,-0.013) | 1024.32  (643.25-1616.87) | -0.239  (-0.369,-0.109) |
| Republic of Seychelles | 243.47  (155.98-355.95) | 1.907  (1.447-2.370) | 151.8  (100.36-214.61) | 0.985  (0.574,1.397) | 1324.42  (1160.74-1474.68) | 0.818  (0.480,1.156) |
| Republic of Sierra Leone | 80.98  (41.74-133.75) | 2.106  (1.986-2.226) | 76.83  (40.11-126.41) | 1.805  (1.675,1.935) | 1011.34  (638.59-1452.6) | 0.112  (-0.050,0.275) |
| Republic of Singapore | 79.7  (57.28-108.8) | 2.319  (2.015-2.624) | 22.38  (17.85-27.54) | -0.618  (-0.863,-0.373) | 383.33  (303.62-473.44) | -0.527  (-0.751,-0.303) |
| Republic of Slovenia | 215.66  (157.66-290.03) | 2.581  (2.105-3.060) | 80  (63.89-97.4) | 0.484  (0.128,0.841) | 1308.63  (1039.5-1621.24) | 0.259  (-0.115,0.635) |
| Republic of South Africa | 162.5  (117.81-205.84) | 1.557  (1.426-1.688) | 126.35  (88.94-157.4) | 0.925  (0.728,1.123) | 1877.08  (1110.89-2947.96) | 0.819  (0.582,1.057) |
| Republic of South Sudan | 72.28  (34.86-126.95) | 0.224  (0.156-0.292) | 66.87  (31.61-116.48) | -0.039  (-0.095,0.016) | 223.25  (133.77-375.55) | -1.562  (-1.780,-1.343) |
| Republic of Sudan | 42.31  (24.61-68.98) | 1.977  (1.843-2.112) | 24.76  (14.65-38.9) | 0.674  (0.603,0.745) | 1469.46  (887.73-2237.31) | 0.838  (0.760,0.917) |
| Republic of Suriname | 171.72  (100.29-269.03) | 1.402  (1.166-1.639) | 109.03  (64.02-171.47) | 0.873  (0.639,1.107) | 872.05  (615.34-1160.6) | 0.692  (0.635,0.749) |
| Republic of Tajikistan | 14.66  (8.54-24.91) | -1.310  (-1.492--1.128) | 11.09  (6.54-19.25) | -1.869  (-2.099,-1.639) | 561.74  (334.05-878.88) | 0.567  (0.539,0.595) |
| Republic of the Congo | 100.05  (54.93-158.18) | 0.610  (0.486-0.735) | 94.86  (52.51-149.13) | 0.200  (0.108,0.292) | 2736.25  (2014.74-3623.04) | -0.581  (-0.824,-0.337) |
| Republic of the Gambia | 23.49  (13.65-37.58) | 1.144  (1.058-1.230) | 21.19  (12.58-33.42) | 0.799  (0.721,0.877) | 255.9  (195.61-326.2) | 0.156  (-0.674,0.993) |
| Republic of the Marshall Islands | 96.33  (58.11-148.45) | 1.153  (1.075-1.230) | 89.77  (54.8-135.62) | 0.879  (0.803,0.956) | 1230.68  (767.11-1869.69) | 0.748  (0.695,0.801) |
| Republic of the Niger | 71.37  (34.2-126.85) | 2.221  (2.072-2.369) | 72.01  (34.77-126.91) | 1.974  (1.840,2.109) | 975.66  (777.6-1201.97) | 1.367  (1.223,1.512) |
| Republic of the Philippines | 65.49  (45.68-89.89) | 1.066  (1.023-1.110) | 48.62  (34.41-64.36) | 0.624  (0.540,0.709) | 1113.5  (971.74-1245.77) | 2.173  (2.038,2.309) |
| Republic of the Union of Myanmar | 40.29  (23.53-64.41) | 1.424  (1.388-1.461) | 33.09  (19.66-51.71) | 0.697  (0.668,0.726) | 1493.33  (750.05-2508.94) | -0.488  (-0.625,-0.351) |
| Republic of Trinidad and Tobago | 320.39  (221.34-445.4) | 0.470  (0.271-0.670) | 157.92  (117.86-204.16) | -0.616  (-0.869,-0.362) | 4523.25  (3331.66-5961.62) | 0.733  (0.527,0.939) |
| Republic of Tunisia | 68.55  (34.67-120.77) | 2.301  (2.250-2.353) | 23.01  (11.98-38.01) | 0.127  (0.015,0.240) | 3574.53  (2739.78-4526.76) | -0.758  (-1.053,-0.462) |
| Republic of Turkey | 154.14  (90.03-240.74) | 2.276  (2.061-2.492) | 48.3  (29.79-71.44) | -1.008  (-1.310,-0.704) | 3943.42  (3276.42-4672.46) | 0.594  (0.309,0.879) |
| Republic of Uganda | 184.9  (115.91-281.44) | 0.799  (0.668-0.930) | 163.3  (104.1-246.8) | 0.394  (0.242,0.545) | 637.47  (365.22-1052.11) | -1.311  (-1.730,-0.890) |
| Republic of Uzbekistan | 19.21  (14.21-25.3) | 0.801  (-0.088-1.697) | 13.15  (10.14-16.56) | 0.272  (-0.536,1.087) | 1157.74  (692.94-1830.14) | 1.135  (0.999,1.271) |
| Republic of Vanuatu | 79.65  (49.53-121.43) | 0.950  (0.902-0.998) | 75.51  (46.87-115.23) | 0.786  (0.723,0.849) | 259.77  (140.57-511.37) | -0.254  (-0.547,0.039) |
| Republic of Yemen | 41.8  (23.44-68.65) | 1.903  (1.797-2.009) | 27.89  (15.79-45.09) | 0.897  (0.845,0.949) | 1671.81  (843.14-2712.62) | 2.140  (2.009,2.271) |
| Republic of Zambia | 148.16  (57.52-270.15) | 2.986  (2.593-3.380) | 124.76  (50.29-221.49) | 2.332  (2.020,2.644) | 2580.36  (1699.4-3656.12) | 0.902  (0.503,1.303) |
| Republic of Zimbabwe | 187.42  (106.97-287.17) | 0.982  (0.658-1.306) | 175.41  (100.01-267.38) | 1.053  (0.756,1.351) | 1315.45  (677.79-2177.81) | 1.807  (1.673,1.941) |
| Romania | 136.99  (101.23-182.24) | 3.567  (3.377-3.757) | 51.25  (41.08-62.85) | 1.259  (1.092,1.426) | 1172.67  (758.57-1713.74) | 0.417  (0.318,0.517) |
| Russian Federation | 179.89  (155.48-201.21) | 4.027  (3.760-4.294) | 55.81  (48.89-61.9) | 2.272  (2.148,2.396) | 191.51  (93.37-308.27) | 0.895  (0.758,1.033) |
| Saint Kitts and Nevis | 501.76  (348.77-697.76) | 2.015  (1.691-2.340) | 280.2  (210.81-363.41) | 0.461  (0.281,0.642) | 1216.01  (692.69-1969.35) | 0.706  (0.655,0.757) |
| Saint Lucia | 406.91  (296.18-540.03) | 0.152  (-0.156-0.461) | 226  (174.52-282.75) | -0.909  (-1.248,-0.569) | 1158.59  (524.39-2195.47) | -0.149  (-0.205,-0.092) |
| Saint Vincent and the Grenadines | 403.27  (316.79-512.22) | 1.094  (0.815-1.373) | 245.08  (205.46-287.27) | 0.801  (0.502,1.101) | 2182.16  (1571.73-2756.22) | 0.968  (0.768,1.169) |
| Slovak Republic | 173.75  (106.18-268.72) | 2.137  (1.990-2.285) | 64.46  (42.02-94.19) | 0.482  (0.383,0.582) | 1235.62  (578.2-2161.6) | -0.121  (-0.199,-0.043) |
| Socialist Republic of Viet Nam | 20.15  (9.54-33.77) | 2.440  (2.310-2.571) | 11.11  (5.32-17.93) | 0.852  (0.705,1.000) | 536.67  (422.35-659.62) | -2.245  (-2.675,-1.814) |
| Solomon Islands | 77.75  (44.8-124.42) | 0.988  (0.943-1.033) | 73.68  (42.2-118.52) | 0.720  (0.690,0.751) | 516.28  (366.56-690.82) | 1.708  (1.052,2.368) |
| State of Eritrea | 84.13  (40.13-140.41) | 0.616  (0.489-0.744) | 81.07  (37.54-135.57) | 0.417  (0.291,0.544) | 420.3  (249.3-665.49) | 0.694  (0.620,0.768) |
| State of Israel | 96.33  (68.99-129.39) | -0.292  (-0.878-0.298) | 32.28  (25.29-39.6) | -2.375  (-2.761,-1.987) | 974.01  (751.53-1204.39) | -2.531  (-2.670,-2.392) |
| State of Kuwait | 132.28  (85.85-190.93) | 3.740  (3.077-4.408) | 29.1  (20.92-38.48) | 1.674  (1.021,2.331) | 499.98  (311.93-805.12) | 0.346  (0.242,0.451) |
| State of Libya | 103.07  (55.1-171.1) | 2.146  (1.912-2.381) | 42.12  (22.84-68.68) | 0.490  (0.396,0.583) | 569.94  (452.32-696.93) | 1.702  (1.324,2.082) |
| State of Qatar | 254.55  (132.51-446.33) | 2.682  (2.320-3.046) | 60.92  (34.59-98.22) | -0.893  (-1.373,-0.411) | 1682.99  (813.86-2783.65) | 2.102  (1.952,2.252) |
| Sultanate of Oman | 47.21  (26.24-79.44) | 2.726  (2.504-2.949) | 13.39  (7.81-22.19) | 0.360  (0.128,0.593) | 1420.36  (899.63-2139.37) | 0.299  (0.247,0.350) |
| Swiss Confederation | 232.46  (164.44-316.03) | -0.960  (-1.225--0.695) | 57.27  (44.38-69.96) | -2.509  (-2.733,-2.285) | 392.23  (205.32-647.78) | 0.289  (0.208,0.370) |
| Syrian Arab Republic | 81.02  (47.49-135.23) | 2.543  (2.368-2.719) | 28.47  (18.06-46) | 0.344  (0.246,0.443) | 843.04  (519.96-1256.43) | -0.692  (-0.950,-0.434) |
| Taiwan (Province of China) | 112.1  (81.34-150.94) | 3.736  (3.126-4.350) | 32.28  (25.75-39.4) | 1.640  (1.268,2.014) | 289.68  (220.72-380.37) | 0.715  (0.392,1.038) |
| Togolese Republic | 105.89  (52.36-173.24) | 2.447  (2.327-2.568) | 97.88  (47.83-160.21) | 2.132  (1.987,2.278) | 1376.79  (878.35-2039.61) | 0.661  (0.590,0.732) |
| Tokelau | 117.21  (72.89-179.31) | 1.146  (1.107-1.185) | 87.38  (55.22-131.28) | 0.314  (0.260,0.368) | 2993.1  (1899.01-4562.8) | 0.339  (0.165,0.513) |
| Turkmenistan | 21.6  (15.74-28.69) | 1.425  (1.062-1.789) | 14.35  (11.05-18.69) | 0.544  (0.237,0.853) | 934.38  (607.8-1359.85) | 0.032  (-0.159,0.224) |
| Tuvalu | 98.37  (62.25-148.4) | 1.263  (1.176-1.351) | 84.67  (54.12-125.9) | 0.704  (0.630,0.778) | 662.28  (407.63-1055.75) | 1.057  (0.524,1.594) |
| Ukraine | 88.23  (55.75-131.57) | 0.630  (0.440-0.821) | 45.4  (30.24-65.1) | -0.196  (-0.441,0.050) | 1130.8  (1026.99-1210.41) | -1.210  (-1.299,-1.121) |
| Union of the Comoros | 86.89  (44.09-145.71) | 0.658  (0.615-0.702) | 79  (40.86-131.86) | 0.317  (0.268,0.365) | 923.24  (769.69-1094.61) | -0.312  (-0.470,-0.155) |
| United Arab Emirates | 94.06  (54.74-154.74) | 2.597  (2.073-3.123) | 39.98  (24.59-63.06) | 1.394  (0.815,1.977) | 1288.2  (612.56-2190.64) | -0.442  (-0.529,-0.355) |
| United Kingdom of Great Britain and Northern Ireland | 227.16  (204.48-247.06) | 0.717  (0.453-0.982) | 68.17  (61.44-72.15) | -1.157  (-1.267,-1.046) | 997.99  (885.38-1093.76) | -2.135  (-2.289,-1.980) |
| United Mexican States | 157.79  (130.14-187.89) | 1.125  (0.823-1.428) | 51.45  (43.31-59.73) | -0.386  (-0.548,-0.223) | 1890.96  (1024.13-3346.68) | -1.571  (-1.965,-1.175) |
| United Republic of Tanzania | 79.95  (38.32-137.88) | -0.022  (-0.113-0.068) | 69.97  (33.74-117.41) | -0.375  (-0.460,-0.290) | 471.3  (266.75-761.66) | 0.869  (0.813,0.925) |
| United States of America | 322.49  (296.09-345.28) | -1.158  (-1.283--1.033) | 49.19  (43.38-52.64) | -2.322  (-2.474,-2.170) | 2482.46  (938.8-4516.97) | 2.578  (2.228,2.928) |
| United States Virgin Islands | 240.03  (125.43-438.65) | -1.065  (-1.501--0.628) | 125.17  (68.77-214.61) | -1.204  (-1.546,-0.861) | 3186.73  (1828.3-4855.14) | 1.160  (0.849,1.471) |

Abbreviations: ASR, age-standardized rate; UI, uncertainty interval; CI, confidence interval; DALYs, disability adjusted life years.

Supplementary table 4 Age trends in the ASIR of prostate cancer in 2021 globally and in different SDI regions.

| Age | Global  (95% CI) | High SDI  (95% CI) | High-middle SDI  (95% CI) | Middle SDI  (95% CI) | Low-middle SDI  (95% CI) | Low SDI  (95% CI) |
| --- | --- | --- | --- | --- | --- | --- |
| 40-44 | 1  (0.86-1.17) | 2.61  (1.99-3.43) | 0.81  (0.73-0.9) | 0.49  (0.45-0.53) | 0.27  (0.25-0.29) | 0.34  (0.3-0.38) |
| 45-49 | 4.25  (3.9-4.62) | 12.51  (10.94-14.31) | 3.1  (2.93-3.28) | 1.65  (1.57-1.74) | 1.1  (1.05-1.14) | 1.51  (1.41-1.61) |
| 50-54 | 16.53  (15.69-17.4) | 49.5  (45.71-53.6) | 11.65  (11.25-12.07) | 5.82  (5.62-6.03) | 4.17  (4.06-4.28) | 5.46  (5.24-5.69) |
| 55-59 | 44.52  (42.91-46.19) | 126.82  (119.88-134.15) | 33.31  (32.49-34.15) | 15.65  (15.25-16.06) | 11.92  (11.7-12.15) | 15.84  (15.39-16.3) |
| 60-64 | 102.95  (100.06-105.92) | 275.86  (264.05-288.2) | 81.87  (80.33-83.44) | 39.37  (38.6-40.16) | 30.34  (29.91-30.78) | 38.93  (38.08-39.8) |
| 65-69 | 181.68  (177.28-186.2) | 448.9  (432.19-466.26) | 152.35  (149.9-154.84) | 75  (73.73-76.29) | 59.32  (58.6-60.06) | 78.75  (77.29-80.24) |
| 70-74 | 267.94  (261.76-274.27) | 592.19  (570.97-614.2) | 235.1  (231.53-238.73) | 125.83  (123.85-127.84) | 101.46  (100.3-102.63) | 128.56  (126.3-130.86) |
| 75-79 | 357.16  (348.63-365.9) | 696.65  (670.52-723.81) | 323.7  (318.64-328.85) | 191.56  (188.54-194.63) | 159.67  (157.83-161.52) | 181.51  (178.21-184.88) |
| 80-84 | 397.3  (386.36-408.54) | 673.5  (644.48-703.83) | 359.56  (352.92-366.33) | 253.06  (248.51-257.69) | 209.76  (207.01-212.55) | 237.04  (232.08-242.11) |
| 85-89 | 476.76  (458.98-495.22) | 713.35  (672.64-756.52) | 436.47  (425.13-448.11) | 356.58  (347.69-365.69) | 280.54  (275.45-285.72) | 314.81  (305.48-324.41) |
| 90-94 | 460.17  (433.35-488.65) | 585.38  (533.13-642.74) | 483.97  (463.87-504.95) | 420.13  (404.06-436.85) | 345.47  (336.23-354.97) | 412.52  (393.73-432.21) |
| 95+ | 427.38  (381-479.4) | 529.26  (444.43-630.3) | 488.74  (448.29-532.84) | 397.37  (369.14-427.75) | 321.06  (304.83-338.15) | 309.67  (276.22-347.18) |

Abbreviations: ASIR, age-standardized incidence rate; SDI, Socio-Demographic Index; CI, confidence interval.

Supplementary table 5 Age trends in the ASMR of prostate cancer in 2021 globally and in different SDI regions.

| Age | Global  (95% CI) | High SDI  (95% CI) | High-middle SDI  (95% CI) | Middle SDI  (95% CI) | Low-middle SDI  (95% CI) | Low SDI  (95% CI) |
| --- | --- | --- | --- | --- | --- | --- |
| 40-44 | 0.28  (0.25-0.31) | 0.32  (0.24-0.45) | 0.34  (0.31-0.38) | 0.25  (0.23-0.27) | 0.16  (0.15-0.18) | 0.27  (0.23-0.31) |
| 45-49 | 0.97  (0.92-1.03) | 1.35  (1.16-1.58) | 1.06  (1-1.12) | 0.76  (0.72-0.8) | 0.64  (0.61-0.69) | 1.17  (1.08-1.27) |
| 50-54 | 3.03  (2.92-3.14) | 4.47  (4.09-4.88) | 3.06  (2.94-3.17) | 2.23  (2.16-2.31) | 2.16  (2.08-2.25) | 3.94  (3.74-4.14) |
| 55-59 | 7.88  (7.69-8.08) | 11.41  (10.78-12.08) | 7.74  (7.55-7.94) | 5.59  (5.46-5.72) | 6.04  (5.88-6.21) | 11.42  (11.03-11.81) |
| 60-64 | 20.41  (20.06-20.77) | 28.53  (27.44-29.66) | 20.08  (19.73-20.44) | 14.71  (14.46-14.95) | 16.36  (16.05-16.68) | 29.45  (28.72-30.19) |
| 65-69 | 40.68  (40.11-41.24) | 53.76  (52.14-55.42) | 39.69  (39.13-40.25) | 29.61  (29.21-30.02) | 34.48  (33.94-35.03) | 63.22  (61.94-64.53) |
| 70-74 | 74.97  (74.06-75.89) | 94.43  (91.98-96.95) | 72.83  (71.93-73.74) | 57.44  (56.76-58.12) | 66.17  (65.25-67.11) | 114.12  (112-116.28) |
| 75-79 | 139.42  (137.85-141.02) | 172.06  (167.97-176.25) | 136.48  (134.92-138.06) | 110.95  (109.74-112.18) | 124.12  (122.48-125.79) | 183.17  (179.8-186.61) |
| 80-84 | 227.13  (224.53-229.76) | 270.1  (263.81-276.55) | 223.17  (220.55-225.81) | 188.26  (186.13-190.41) | 194.19  (191.46-196.95) | 271.52  (266.06-277.1) |
| 85-89 | 367.52  (362.32-372.8) | 411.91  (400.49-423.65) | 365.3  (359.91-370.76) | 326.61  (321.87-331.43) | 299.98  (294.48-305.57) | 398.17  (387.27-409.39) |
| 90-94 | 535.55  (526.09-545.18) | 572.24  (553.53-591.59) | 544.37  (533.95-554.98) | 487.9  (478.63-497.34) | 439.47  (428.85-450.35) | 582.67  (559.82-606.46) |
| 95+ | 538.7  (523.01-554.87) | 561.41  (533.34-590.96) | 590.67  (570.75-611.29) | 491.64  (475.91-507.88) | 439.73  (421.28-458.98) | 478.2  (436.47-523.93) |

Abbreviations: ASMR, age-standardized mortality rate; SDI, Socio-Demographic Index; CI, confidence interval.

Supplementary table 6 Age trends in the age-standardized DALY rate of prostate cancer in 2021 globally and in different SDI regions.

| Age | Global  (95% CI) | High SDI  (95% CI) | High-middle SDI  (95% CI) | Middle SDI  (95% CI) | Low-middle SDI  (95% CI) | Low SDI  (95% CI) |
| --- | --- | --- | --- | --- | --- | --- |
| 40-44 | 13.89  (13.03-14.82) | 16.63  (13.19-20.96) | 16.77  (15.74-17.86) | 12.31  (11.63-13.02) | 8.15  (7.93-8.39) | 13.08  (12.19-14.03) |
| 45-49 | 44.24  (42.56-45.99) | 64.81  (57.49-73.05) | 47.26  (45.45-49.14) | 33.89  (32.65-35.17) | 28.44  (27.95-28.94) | 50.68  (48.6-52.84) |
| 50-54 | 126.09  (122.87-129.39) | 202.5  (188.15-217.93) | 124.81  (121.57-128.14) | 89.37  (87.06-91.75) | 85.04  (84.03-86.07) | 152.26  (147.96-156.69) |
| 55-59 | 293.5  (288.08-299.02) | 467.86  (444.59-492.33) | 283.47  (278.18-288.85) | 198.03  (194.17-201.97) | 209.38  (207.54-211.23) | 389.04  (381.06-397.18) |
| 60-64 | 657.8  (648.51-667.23) | 1010.54  (972.61-1049.95) | 638.43  (629.42-647.58) | 450.68  (443.83-457.64) | 490.05  (486.73-493.39) | 867.97  (853.94-882.23) |
| 65-69 | 1109.45  (1095.85-1123.22) | 1609.31  (1557.49-1662.84) | 1071.98  (1058.85-1085.27) | 767.81  (757.57-778.19) | 872.53  (867.37-877.73) | 1575.31  (1552.89-1598.06) |
| 70-74 | 1674.07  (1654.73-1693.64) | 2283.61  (2214.85-2354.51) | 1615.93  (1597.21-1634.87) | 1225.77  (1210.48-1241.24) | 1379.42  (1371.67-1387.22) | 2343.1  (2310.63-2376.02) |
| 75-79 | 2459.63  (2431.32-2488.26) | 3225.69  (3130.14-3324.17) | 2396.35  (2368.78-2424.24) | 1885.15  (1861.82-1908.78) | 2064.75  (2052.97-2076.59) | 3006.08  (2961.87-3050.96) |
| 80-84 | 3096.5  (3057.46-3136.04) | 3867.43  (3746.76-3991.98) | 3019.73  (2981.2-3058.75) | 2488.7  (2454.21-2523.67) | 2523.21  (2506.62-2539.92) | 3484.56  (3423.05-3547.18) |
| 85-89 | 3913.2  (3848.21-3979.28) | 4573.22  (4394.16-4759.58) | 3853.51  (3787.24-3920.93) | 3391.7  (3327.36-3457.29) | 3077.22  (3048.76-3105.95) | 4044.14  (3938.46-4152.67) |
| 90-94 | 4801.58  (4690.08-4915.74) | 5257  (4985.69-5543.08) | 4914.77  (4791.57-5041.14) | 4357.58  (4236.68-4481.93) | 3884.57  (3831.71-3938.15) | 5095.97  (4883.17-5318.04) |
| 95+ | 4473.72  (4283.78-4672.08) | 4781.93  (4359.37-5245.44) | 5003.03  (4759.95-5258.54) | 4021.13  (3813.89-4239.64) | 3567.28  (3474.29-3662.77) | 3953.93  (3561-4390.22) |

Abbreviations: SDI, Socio-Demographic Index; CI, confidence interval; DALYs, disability adjusted life years.

Supplementary table 7 Period trends in the rate ratio of ASIR of prostate cancer in 2021 globally and in different SDI regions.

| Period | Global  (95% CI) | High SDI  (95% CI) | High-middle SDI  (95% CI) | Middle SDI  (95% CI) | Low-middle SDI  (95% CI) | Low SDI  (95% CI) |
| --- | --- | --- | --- | --- | --- | --- |
| 1992-1996 | 0.89  (0.86-0.92) | 0.93  (0.89-0.98) | 0.77  (0.75-0.78) | 0.73  (0.71-0.74) | 0.71  (0.7-0.72) | 0.89  (0.87-0.92) |
| 1997-2001 | 0.95  (0.93-0.98) | 0.98  (0.94-1.02) | 0.88  (0.87-0.9) | 0.88  (0.86-0.9) | 0.83  (0.81-0.84) | 0.93  (0.9-0.95) |
| 2002-2006 | 0.99  (0.97-1.02) | 1.01  (0.97-1.05) | 0.96  (0.94-0.97) | 0.96  (0.94-0.97) | 0.91  (0.9-0.92) | 0.96  (0.94-0.98) |
| 2007-2011 | 1  (1-1) | 1  (1-1) | 1  (1-1) | 1  (1-1) | 1  (1-1) | 1  (1-1) |
| 2012-2016 | 0.93  (0.9-0.95) | 0.89  (0.86-0.92) | 0.97  (0.95-0.98) | 1.01  (0.99-1.03) | 1.05  (1.04-1.06) | 1.05  (1.03-1.07) |
| 2017-2021 | 0.89  (0.86-0.91) | 0.83  (0.8-0.87) | 0.95  (0.93-0.96) | 1.04  (1.02-1.05) | 1.1  (1.09-1.11) | 1.12  (1.1-1.14) |

Abbreviations: ASIR, age-standardized incidence rate; SDI, Socio-Demographic Index; CI, confidence interval.

Supplementary table 8 Period trends in the rate ratio of ASMR of prostate cancer in 2021 globally and in different SDI regions.

| Period | Global  (95% CI) | High SDI  (95% CI) | High-middle SDI  (95% CI) | Middle SDI  (95% CI) | Low-middle SDI  (95% CI) | Low SDI  (95% CI) |
| --- | --- | --- | --- | --- | --- | --- |
| 1992-1996 | 1.15  (1.13-1.17) | 1.42  (1.37-1.48) | 1.13  (1.11-1.15) | 0.96  (0.95-0.98) | 0.88  (0.87-0.9) | 1  (0.97-1.02) |
| 1997-2001 | 1.12  (1.1-1.13) | 1.26  (1.22-1.3) | 1.13  (1.12-1.15) | 1.02  (1.01-1.04) | 0.94  (0.93-0.96) | 0.99  (0.97-1.02) |
| 2002-2006 | 1.07  (1.05-1.08) | 1.13  (1.1-1.16) | 1.08  (1.07-1.1) | 1.03  (1.02-1.04) | 0.97  (0.96-0.99) | 0.99  (0.97-1.01) |
| 2007-2011 | 1  (1-1) | 1  (1-1) | 1  (1-1) | 1  (1-1) | 1  (1-1) | 1  (1-1) |
| 2012-2016 | 0.93  (0.92-0.95) | 0.89  (0.87-0.91) | 0.93  (0.92-0.94) | 0.95  (0.94-0.96) | 1.01  (1-1.02) | 1.04  (1.02-1.06) |
| 2017-2021 | 0.91  (0.9-0.92) | 0.86  (0.84-0.89) | 0.88  (0.87-0.89) | 0.91  (0.9-0.92) | 1.03  (1.02-1.05) | 1.08  (1.06-1.1) |

Abbreviations: ASMR, age-standardized mortality rate; SDI, Socio-Demographic Index; CI, confidence interval.

Supplementary table 9 Period trends in the rate ratio of age-standardized DALY rate of prostate cancer in 2021 globally and in different SDI regions.

| Period | Global  (95% CI) | High SDI  (95% CI) | High-middle SDI  (95% CI) | Middle SDI  (95% CI) | Low-middle SDI  (95% CI) | Low SDI  (95% CI) |
| --- | --- | --- | --- | --- | --- | --- |
| 1992-1996 | 1.11  (1.1-1.13) | 1.29  (1.24-1.34) | 1.1  (1.08-1.12) | 0.96  (0.94-0.97) | 0.88  (0.87-0.88) | 0.98  (0.96-1) |
| 1997-2001 | 1.1  (1.08-1.11) | 1.19  (1.15-1.23) | 1.11  (1.1-1.13) | 1.02  (1.01-1.04) | 0.94  (0.93-0.94) | 0.99  (0.97-1) |
| 2002-2006 | 1.06  (1.05-1.07) | 1.1  (1.07-1.14) | 1.07  (1.06-1.09) | 1.03  (1.01-1.04) | 0.97  (0.97-0.98) | 0.99  (0.97-1) |
| 2007-2011 | 1  (1-1) | 1  (1-1) | 1  (1-1) | 1  (1-1) | 1  (1-1) | 1  (1-1) |
| 2012-2016 | 0.93  (0.92-0.94) | 0.89  (0.86-0.91) | 0.94  (0.92-0.95) | 0.96  (0.94-0.97) | 1.01  (1.01-1.02) | 1.03  (1.02-1.05) |
| 2017-2021 | 0.9  (0.89-0.91) | 0.85  (0.82-0.87) | 0.89  (0.88-0.9) | 0.93  (0.92-0.94) | 1.04  (1.03-1.04) | 1.07  (1.05-1.09) |

Abbreviations: SDI, Socio-Demographic Index; CI, confidence interval; DALYs, disability adjusted life years.

Supplementary table 10 Cohort trends in the rate ratio of ASIR of prostate cancer in 2021 globally and in different SDI regions.

| Cohort | Global  (95% CI) | High SDI  (95% CI) | High-middle SDI  (95% CI) | Middle SDI  (95% CI) | Low-middle SDI  (95% CI) | Low SDI  (95% CI) |
| --- | --- | --- | --- | --- | --- | --- |
| 1892-1901 | 1.46  (0.98-2.17) | 1.88  (1.06-3.35) | 1.07  (0.77-1.47) | 0.93  (0.7-1.24) | 0.6  (0.49-0.74) | 0.75  (0.5-1.13) |
| 1897-1906 | 1.34  (1.14-1.58) | 1.7  (1.34-2.16) | 1.04  (0.92-1.17) | 0.91  (0.82-1.02) | 0.64  (0.59-0.69) | 0.76  (0.66-0.87) |
| 1902-1911 | 1.3  (1.2-1.41) | 1.58  (1.4-1.79) | 1.02  (0.96-1.08) | 0.89  (0.84-0.95) | 0.67  (0.65-0.7) | 0.78  (0.72-0.83) |
| 1907-1916 | 1.29  (1.22-1.36) | 1.49  (1.38-1.61) | 1.03  (1-1.07) | 0.89  (0.86-0.93) | 0.75  (0.73-0.77) | 0.84  (0.8-0.87) |
| 1912-1921 | 1.18  (1.13-1.23) | 1.35  (1.27-1.43) | 0.95  (0.92-0.98) | 0.87  (0.85-0.9) | 0.81  (0.8-0.83) | 0.87  (0.85-0.9) |
| 1917-1926 | 1.13  (1.09-1.17) | 1.22  (1.16-1.28) | 0.93  (0.91-0.95) | 0.87  (0.85-0.89) | 0.86  (0.85-0.87) | 0.92  (0.9-0.94) |
| 1922-1931 | 1.05  (1.02-1.08) | 1.09  (1.05-1.14) | 0.94  (0.92-0.96) | 0.89  (0.87-0.9) | 0.9  (0.89-0.91) | 0.95  (0.93-0.97) |
| 1927-1936 | 1  (0.97-1.03) | 1.03  (0.98-1.07) | 0.95  (0.94-0.97) | 0.92  (0.91-0.94) | 0.93  (0.92-0.95) | 0.98  (0.96-1) |
| 1932-1941 | 1  (1-1) | 1  (1-1) | 1  (1-1) | 1  (1-1) | 1  (1-1) | 1  (1-1) |
| 1937-1946 | 1.04  (1.01-1.07) | 1.02  (0.98-1.06) | 1.06  (1.05-1.08) | 1.11  (1.09-1.13) | 1.09  (1.07-1.1) | 1  (0.98-1.02) |
| 1942-1951 | 1.09  (1.06-1.12) | 1.03  (0.99-1.08) | 1.14  (1.11-1.16) | 1.21  (1.18-1.23) | 1.23  (1.21-1.25) | 1.03  (1.01-1.06) |
| 1947-1956 | 1.13  (1.09-1.17) | 1.09  (1.04-1.15) | 1.22  (1.2-1.25) | 1.31  (1.29-1.34) | 1.39  (1.37-1.42) | 1.1  (1.08-1.13) |
| 1952-1961 | 1.21  (1.16-1.26) | 1.15  (1.08-1.23) | 1.39  (1.35-1.43) | 1.54  (1.5-1.59) | 1.58  (1.55-1.62) | 1.22  (1.18-1.26) |
| 1957-1966 | 1.22  (1.16-1.29) | 1.18  (1.08-1.29) | 1.48  (1.43-1.54) | 1.69  (1.63-1.75) | 1.76  (1.71-1.81) | 1.33  (1.27-1.39) |
| 1962-1971 | 1.18  (1.08-1.29) | 1.16  (1.01-1.34) | 1.5  (1.41-1.59) | 1.81  (1.71-1.9) | 1.96  (1.88-2.04) | 1.42  (1.33-1.51) |
| 1967-1976 | 1.23  (1.05-1.44) | 1.12  (0.85-1.49) | 1.69  (1.53-1.88) | 2.04  (1.87-2.22) | 2.22  (2.08-2.37) | 1.53  (1.38-1.7) |
| 1972-1981 | 1.42  (1.02-1.96) | 1.19  (0.63-2.26) | 2.14  (1.75-2.62) | 2.43  (2.07-2.84) | 2.56  (2.27-2.9) | 1.69  (1.38-2.07) |

Abbreviations: ASIR, age-standardized incidence rate; SDI, Socio-Demographic Index; CI, confidence interval.

Supplementary table 11 Cohort trends in the rate ratio of ASMR of prostate cancer in 2021 globally and in different SDI regions.

| Cohort | Global  (95% CI) | High SDI  (95% CI) | High-middle SDI  (95% CI) | Middle SDI  (95% CI) | Low-middle SDI  (95% CI) | Low SDI  (95% CI) |
| --- | --- | --- | --- | --- | --- | --- |
| 1892-1901 | 1.52  (1.38-1.67) | 2.12  (1.82-2.48) | 1.31  (1.16-1.48) | 1.14  (1.01-1.28) | 0.67  (0.57-0.79) | 0.77  (0.56-1.07) |
| 1897-1906 | 1.42  (1.37-1.48) | 1.96  (1.83-2.1) | 1.27  (1.21-1.33) | 1.1  (1.05-1.15) | 0.72  (0.67-0.76) | 0.78  (0.7-0.88) |
| 1902-1911 | 1.39  (1.36-1.43) | 1.86  (1.78-1.95) | 1.27  (1.24-1.31) | 1.1  (1.07-1.13) | 0.77  (0.74-0.8) | 0.8  (0.75-0.85) |
| 1907-1916 | 1.4  (1.38-1.43) | 1.8  (1.74-1.86) | 1.31  (1.28-1.34) | 1.11  (1.08-1.13) | 0.86  (0.84-0.89) | 0.86  (0.83-0.9) |
| 1912-1921 | 1.31  (1.29-1.33) | 1.64  (1.59-1.69) | 1.23  (1.21-1.25) | 1.08  (1.06-1.09) | 0.93  (0.91-0.95) | 0.91  (0.88-0.93) |
| 1917-1926 | 1.24  (1.23-1.26) | 1.49  (1.45-1.53) | 1.16  (1.14-1.18) | 1.05  (1.04-1.07) | 0.97  (0.96-0.99) | 0.96  (0.93-0.98) |
| 1922-1931 | 1.15  (1.14-1.17) | 1.31  (1.28-1.35) | 1.11  (1.09-1.12) | 1.02  (1.01-1.04) | 0.99  (0.98-1.01) | 0.99  (0.97-1.01) |
| 1927-1936 | 1.06  (1.05-1.08) | 1.15  (1.12-1.18) | 1.04  (1.03-1.05) | 1  (0.99-1.01) | 0.99  (0.98-1.01) | 1.01  (0.99-1.03) |
| 1932-1941 | 1  (1-1) | 1  (1-1) | 1  (1-1) | 1  (1-1) | 1  (1-1) | 1  (1-1) |
| 1937-1946 | 0.96  (0.94-0.97) | 0.9  (0.87-0.93) | 0.95  (0.94-0.96) | 1  (0.99-1.02) | 1.01  (0.99-1.03) | 0.97  (0.94-0.99) |
| 1942-1951 | 0.91  (0.89-0.92) | 0.81  (0.78-0.84) | 0.88  (0.87-0.9) | 0.97  (0.96-0.99) | 1.04  (1.02-1.06) | 0.95  (0.93-0.97) |
| 1947-1956 | 0.87  (0.85-0.89) | 0.76  (0.72-0.79) | 0.85  (0.83-0.86) | 0.95  (0.93-0.97) | 1.06  (1.04-1.09) | 0.96  (0.94-0.99) |
| 1952-1961 | 0.86  (0.84-0.88) | 0.7  (0.65-0.74) | 0.82  (0.8-0.84) | 0.98  (0.96-1.01) | 1.1  (1.06-1.13) | 1.01  (0.97-1.05) |
| 1957-1966 | 0.81  (0.77-0.84) | 0.62  (0.56-0.69) | 0.73  (0.7-0.76) | 0.94  (0.91-0.98) | 1.12  (1.07-1.17) | 1.03  (0.97-1.08) |
| 1962-1971 | 0.75  (0.7-0.8) | 0.55  (0.45-0.66) | 0.61  (0.57-0.66) | 0.89  (0.84-0.94) | 1.15  (1.08-1.23) | 1.03  (0.95-1.12) |
| 1967-1976 | 0.74  (0.66-0.83) | 0.48  (0.33-0.69) | 0.58  (0.51-0.66) | 0.9  (0.82-0.98) | 1.22  (1.09-1.36) | 1.05  (0.91-1.2) |
| 1972-1981 | 0.76  (0.61-0.96) | 0.45  (0.2-1.03) | 0.6  (0.47-0.78) | 0.94  (0.79-1.12) | 1.29  (1.04-1.59) | 1.09  (0.83-1.42) |

Abbreviations: ASMR, age-standardized mortality rate; SDI, Socio-Demographic Index; CI, confidence interval.

Supplementary table 12 Cohort trends in the rate ratio of age-standardized DALY rate of prostate cancer in 2021 globally and in different SDI regions.

| Cohort | Global  (95% CI) | High SDI  (95% CI) | High-middle SDI  (95% CI) | Middle SDI  (95% CI) | Low-middle SDI  (95% CI) | Low SDI  (95% CI) |
| --- | --- | --- | --- | --- | --- | --- |
| 1892-1901 | 1.55  (1.34-1.79) | 2.15  (1.6-2.88) | 1.29  (1.07-1.54) | 1.13  (0.93-1.38) | 0.68  (0.61-0.75) | 0.78  (0.54-1.14) |
| 1897-1906 | 1.42  (1.34-1.51) | 1.95  (1.72-2.21) | 1.24  (1.16-1.33) | 1.08  (1-1.17) | 0.71  (0.69-0.74) | 0.79  (0.7-0.9) |
| 1902-1911 | 1.39  (1.35-1.44) | 1.84  (1.71-1.98) | 1.24  (1.2-1.29) | 1.08  (1.04-1.13) | 0.76  (0.75-0.78) | 0.81  (0.76-0.86) |
| 1907-1916 | 1.4  (1.37-1.43) | 1.77  (1.68-1.86) | 1.28  (1.25-1.31) | 1.09  (1.06-1.12) | 0.86  (0.85-0.87) | 0.87  (0.84-0.91) |
| 1912-1921 | 1.3  (1.27-1.32) | 1.61  (1.54-1.68) | 1.19  (1.17-1.22) | 1.06  (1.04-1.08) | 0.93  (0.92-0.93) | 0.92  (0.89-0.94) |
| 1917-1926 | 1.23  (1.22-1.25) | 1.46  (1.4-1.51) | 1.14  (1.12-1.16) | 1.04  (1.02-1.05) | 0.97  (0.96-0.98) | 0.96  (0.94-0.98) |
| 1922-1931 | 1.14  (1.12-1.15) | 1.27  (1.23-1.31) | 1.09  (1.08-1.11) | 1.01  (1-1.03) | 0.99  (0.98-0.99) | 0.99  (0.97-1) |
| 1927-1936 | 1.05  (1.04-1.07) | 1.12  (1.08-1.16) | 1.03  (1.01-1.04) | 0.99  (0.98-1.01) | 0.99  (0.98-0.99) | 1  (0.99-1.02) |
| 1932-1941 | 1  (1-1) | 1  (1-1) | 1  (1-1) | 1  (1-1) | 1  (1-1) | 1  (1-1) |
| 1937-1946 | 0.97  (0.95-0.98) | 0.93  (0.89-0.96) | 0.95  (0.94-0.97) | 1.01  (0.99-1.02) | 1.01  (1.01-1.02) | 0.97  (0.95-0.98) |
| 1942-1951 | 0.93  (0.92-0.94) | 0.86  (0.83-0.89) | 0.9  (0.89-0.92) | 0.99  (0.97-1) | 1.05  (1.04-1.06) | 0.96  (0.94-0.97) |
| 1947-1956 | 0.9  (0.88-0.92) | 0.82  (0.79-0.86) | 0.87  (0.86-0.89) | 0.96  (0.95-0.98) | 1.08  (1.07-1.09) | 0.98  (0.96-0.99) |
| 1952-1961 | 0.9  (0.88-0.92) | 0.79  (0.74-0.83) | 0.86  (0.84-0.88) | 1  (0.98-1.02) | 1.12  (1.11-1.13) | 1.02  (1-1.05) |
| 1957-1966 | 0.85  (0.82-0.88) | 0.72  (0.66-0.79) | 0.77  (0.75-0.8) | 0.97  (0.94-1) | 1.14  (1.13-1.16) | 1.05  (1.02-1.08) |
| 1962-1971 | 0.79  (0.76-0.83) | 0.65  (0.57-0.75) | 0.66  (0.63-0.69) | 0.92  (0.88-0.96) | 1.18  (1.16-1.2) | 1.05  (1.01-1.1) |
| 1967-1976 | 0.79  (0.73-0.85) | 0.58  (0.45-0.76) | 0.63  (0.58-0.68) | 0.93  (0.87-0.99) | 1.25  (1.21-1.29) | 1.08  (1.01-1.16) |
| 1972-1981 | 0.82  (0.71-0.94) | 0.57  (0.32-1.02) | 0.67  (0.57-0.77) | 0.97  (0.86-1.09) | 1.32  (1.25-1.4) | 1.12  (0.98-1.28) |

Abbreviations: SDI, Socio-Demographic Index; CI, confidence interval; DALYs, disability adjusted life years.

Supplementary table 13 Trends and projections in the counts and ASR of incidence, mortality, and DALYs for prostate cancer globally from 1990 to 2040.

| Year | Incidence | | Mortality | | DALYs | |
| --- | --- | --- | --- | --- | --- | --- |
|  | Counts  (95% CI) | Rate  (95% CI) | Counts  (95% CI) | Rate  (95% CI) | Counts  (95% CI) | Rate  (95% CI) |
| 1990 | 575728.64  (573527.39-577929.89) | 41.04  (40.92-41.16) | 268086.86  (266544.14-269629.58) | 19.11  (19.02-19.19) | 4780013.29  (4773610.26-4786416.31) | 340.77  (340.43-341.11) |
| 1991 | 604052.81  (601802.77-606302.86) | 42.02  (41.9-42.13) | 277055.13  (275502.64-278607.62) | 19.27  (19.19-19.35) | 4942072.01  (4935572.18-4948571.83) | 343.81  (343.48-344.15) |
| 1992 | 635220.62  (632915.26-637525.99) | 43.13  (43.02-43.25) | 286980.83  (285403.74-288557.92) | 19.48  (19.4-19.56) | 5119367.88  (5112757.88-5125977.88) | 347.67  (347.33-348) |
| 1993 | 667138.5  (664777-669500) | 44.22  (44.11-44.34) | 296744.96  (295142.46-298347.47) | 19.67  (19.59-19.75) | 5294303.87  (5287585.07-5301022.67) | 350.97  (350.64-351.3) |
| 1994 | 701359.21  (698938.97-703779.46) | 45.38  (45.27-45.5) | 306477.7  (304850.04-308105.36) | 19.83  (19.75-19.91) | 5471576.69  (5464749.81-5478403.57) | 354.06  (353.74-354.39) |
| 1995 | 732661.19  (730188.49-735133.88) | 46.27  (46.16-46.39) | 313643.16  (311997.39-315288.92) | 19.81  (19.73-19.88) | 5602435.66  (5595530.09-5609341.24) | 353.87  (353.55-354.2) |
| 1996 | 762193.62  (759672.84-764714.4) | 47.01  (46.9-47.13) | 320566.81  (318903.51-322230.1) | 19.77  (19.69-19.85) | 5727174.23  (5720195.53-5734152.94) | 353.3  (352.98-353.62) |
| 1997 | 779221.09  (776673.99-781768.19) | 46.98  (46.86-47.09) | 325408.65  (323734.34-327082.96) | 19.61  (19.54-19.69) | 5809415.04  (5802392.26-5816437.81) | 350.25  (349.94-350.57) |
| 1998 | 800488.85  (797909.84-803067.85) | 47.2  (47.09-47.31) | 331353.34  (329665.97-333040.71) | 19.54  (19.46-19.61) | 5920734.81  (5913653.75-5927815.87) | 349.14  (348.83-349.45) |
| 1999 | 818172.53  (815568.22-820776.85) | 47.2  (47.09-47.31) | 336154.38  (334457.3-337851.46) | 19.39  (19.32-19.46) | 6008539.62  (6001415.81-6015663.42) | 346.65  (346.35-346.96) |
| 2000 | 836515.34  (833884.76-839145.93) | 47.18  (47.08-47.29) | 341854.81  (340146.02-343563.61) | 19.28  (19.21-19.35) | 6112170.63  (6104995.35-6119345.91) | 344.8  (344.5-345.1) |
| 2001 | 862309.89  (859641.64-864978.14) | 47.51  (47.41-47.62) | 348380.45  (346657.38-350103.51) | 19.19  (19.12-19.26) | 6232358.26  (6225120.89-6239595.64) | 343.44  (343.14-343.73) |
| 2002 | 891302.34  (888591.7-894012.99) | 47.92  (47.81-48.02) | 354732.02  (352994.71-356469.33) | 19.07  (19-19.14) | 6356015.3  (6348713.04-6363317.57) | 341.73  (341.44-342.02) |
| 2003 | 920305.02  (917551.21-923058.83) | 48.2  (48.1-48.31) | 361066.94  (359314.74-362819.13) | 18.91  (18.84-18.98) | 6476296.25  (6468927.29-6483665.21) | 339.25  (338.97-339.54) |
| 2004 | 948669.44  (945874.16-951464.71) | 48.38  (48.28-48.49) | 365949.45  (364186.41-367712.5) | 18.66  (18.59-18.73) | 6572684.49  (6565263.36-6580105.61) | 335.23  (334.95-335.51) |
| 2005 | 984569.6  (981722.92-987416.29) | 48.88  (48.78-48.98) | 370078.3  (368307.19-371849.41) | 18.37  (18.3-18.43) | 6666838.27  (6659368.14-6674308.39) | 331.01  (330.74-331.29) |
| 2006 | 1016891.88  (1014001.23-1019782.52) | 49.16  (49.06-49.26) | 374574.93  (372795.93-376353.93) | 18.11  (18.04-18.17) | 6754370.61  (6746858.78-6761882.44) | 326.57  (326.31-326.84) |
| 2007 | 1046414.35  (1043485.14-1049343.57) | 49.26  (49.16-49.36) | 380317.44  (378528.02-382106.86) | 17.9  (17.84-17.96) | 6854499.91  (6846941.81-6862058.01) | 322.72  (322.46-322.98) |
| 2008 | 1074620.03  (1071654.66-1077585.4) | 49.28  (49.18-49.38) | 386583.3  (384782.36-388384.23) | 17.72  (17.66-17.79) | 6973770.66  (6966156.88-6981384.44) | 319.82  (319.57-320.08) |
| 2009 | 1096418.57  (1093427.19-1099409.95) | 48.99  (48.9-49.09) | 391330.67  (389522.52-393138.82) | 17.48  (17.42-17.54) | 7064196.36  (7056545.85-7071846.87) | 315.7  (315.45-315.95) |
| 2010 | 1118246.41  (1115228.74-1121264.08) | 48.72  (48.62-48.81) | 396385.24  (394569.7-398200.78) | 17.27  (17.21-17.32) | 7165185.25  (7157492.7-7172877.81) | 312.19  (311.94-312.43) |
| 2011 | 1134480.97  (1131446.03-1137515.91) | 48.21  (48.12-48.31) | 401632.44  (399809.51-403455.38) | 17.06  (17.01-17.12) | 7259864  (7252135.53-7267592.46) | 308.55  (308.31-308.79) |
| 2012 | 1143191.04  (1140150.59-1146231.5) | 47.41  (47.32-47.5) | 404494.79  (402670.55-406319.04) | 16.77  (16.72-16.83) | 7314924.15  (7307184.72-7322663.57) | 303.39  (303.16-303.63) |
| 2013 | 1147374.25  (1144333.94-1150414.56) | 46.47  (46.38-46.56) | 407206.23  (405380.59-409031.87) | 16.49  (16.43-16.54) | 7352316.49  (7344573.95-7360059.04) | 297.79  (297.56-298.02) |
| 2014 | 1173586.9  (1170518.8-1176654.99) | 46.46  (46.37-46.54) | 415345.19  (413505.86-417184.52) | 16.44  (16.39-16.49) | 7491233.36  (7483435.95-7499030.78) | 296.57  (296.34-296.79) |
| 2015 | 1199914.69  (1196818.34-1203011.04) | 46.47  (46.38-46.56) | 424674.01  (422818.17-426529.84) | 16.44  (16.39-16.5) | 7640561.4  (7632703.19-7648419.61) | 295.93  (295.71-296.15) |
| 2016 | 1231763.33  (1228634.17-1234892.5) | 46.71  (46.62-46.8) | 434856.64  (432983.6-436729.68) | 16.49  (16.44-16.54) | 7815341.51  (7807415.36-7823267.65) | 296.4  (296.18-296.61) |
| 2017 | 1253002.18  (1249855.7-1256148.67) | 46.56  (46.47-46.64) | 440007.91  (438130.1-441885.72) | 16.35  (16.29-16.4) | 7921448.95  (7913494.96-7929402.95) | 294.35  (294.14-294.56) |
| 2018 | 1279547.34  (1276376.96-1282717.71) | 46.6  (46.51-46.68) | 447469.29  (445581.46-449357.13) | 16.29  (16.24-16.34) | 8058663.9  (8050665.88-8066661.92) | 293.49  (293.28-293.7) |
| 2019 | 1304965.52  (1301773.32-1308157.72) | 46.58  (46.5-46.66) | 455732.15  (453832.84-457631.45) | 16.26  (16.22-16.31) | 8202700.46  (8194656.46-8210744.45) | 292.83  (292.62-293.03) |
| 2020 | 1318019.04  (1314818.04-1321220.03) | 46.16  (46.08-46.24) | 459776.77  (457873.28-461680.26) | 16.1  (16.05-16.15) | 8285788.44  (8277722.5-8293854.37) | 290.22  (290.02-290.42) |
| 2021 | 1337219.74  (1334001.41-1340438.07) | 46.03  (45.95-46.11) | 461267.75  (459358.88-463176.62) | 15.88  (15.83-15.92) | 8345326.14  (8337246.77-8353405.5) | 287.3  (287.1-287.5) |
| 2022 | 1436448.08  (1368210.34-1504685.83) | 47.89  (45.61-50.16) | 474391.39  (463738.6-485044.17) | 15.81  (15.46-16.16) | 8645759.98  (8432105.9-8859414.07) | 288.25  (281.13-295.37) |
| 2023 | 1482959.09  (1393755.81-1572162.36) | 48.45  (45.54-51.37) | 480944.21  (467271.48-494616.93) | 15.71  (15.27-16.16) | 8788653.22  (8513885.75-9063420.68) | 287.18  (278.2-296.15) |
| 2024 | 1531143.88  (1422265.8-1640021.97) | 49.02  (45.53-52.5) | 487786.45  (471410.61-504162.29) | 15.61  (15.09-16.13) | 8936561.59  (8606601.27-9266521.92) | 286.11  (275.55-296.68) |
| 2025 | 1580754.34  (1452601.22-1708907.45) | 49.59  (45.57-53.61) | 494724.52  (475808.07-513640.97) | 15.52  (14.92-16.11) | 9087255.46  (8705038.1-9469472.83) | 285.08  (273.09-297.07) |
| 2026 | 1631588.3  (1484131.26-1779045.34) | 50.17  (45.63-54.7) | 501669.33  (480291.36-523047.29) | 15.42  (14.77-16.08) | 9239566.2  (8806483.77-9672648.62) | 284.12  (270.81-297.44) |
| 2027 | 1682904.42  (1515912.55-1849896.29) | 50.75  (45.72-55.79) | 508436.09  (484630.65-532241.54) | 15.33  (14.61-16.05) | 9389170.02  (8905829.38-9872510.67) | 283.19  (268.61-297.77) |
| 2028 | 1734681.54  (1547834.09-1921529) | 51.33  (45.8-56.86) | 515109.62  (488920.78-541298.47) | 15.24  (14.47-16.01) | 9536941.6  (9003948.55-10069934.65) | 282.23  (266.46-298) |
| 2029 | 1787488.72  (1580300.08-1994677.35) | 51.9  (45.89-57.92) | 521836.21  (493288.71-550383.72) | 15.15  (14.32-15.98) | 9686316.51  (9103823.86-10268809.16) | 281.28  (264.37-298.2) |
| 2030 | 1841078.73  (1613000.54-2069156.91) | 52.48  (45.98-58.99) | 528484.7  (497591.14-559378.25) | 15.06  (14.18-15.94) | 9835429.33  (9203387.63-10467471.03) | 280.4  (262.38-298.42) |
| 2031 | 1895232.59  (1645670.23-2144794.95) | 53.08  (46.09-60.07) | 534927.35  (501689.24-568165.46) | 14.98  (14.05-15.91) | 9982544.69  (9300701.05-10664388.32) | 279.63  (260.53-298.73) |
| 2032 | 1949134.1  (1677525.26-2220742.94) | 53.7  (46.22-61.19) | 540897.47  (505316.43-576478.5) | 14.9  (13.92-15.88) | 10122281.43  (9390411.75-10854151.1) | 278.91  (258.75-299.08) |
| 2033 | 2002377.45  (1708233.46-2296521.45) | 54.33  (46.35-62.31) | 546309  (508417.99-584200.02) | 14.82  (13.79-15.85) | 10252844.08  (9471179.31-11034508.85) | 278.19  (256.98-299.4) |
| 2034 | 2055879.31  (1738552.5-2373206.12) | 54.96  (46.48-63.45) | 551431.08  (511246.45-591615.71) | 14.74  (13.66-15.81) | 10379687.07  (9548052.07-11211322.07) | 277.51  (255.28-299.75) |
| 2035 | 2110310.52  (1768995.35-2451625.69) | 55.62  (46.63-64.62) | 556417.4  (513936.12-598898.69) | 14.66  (13.54-15.78) | 10505436.43  (9623298.62-11387574.24) | 276.93  (253.67-300.18) |
| 2036 | 2166392.79  (1800080.57-2532705.01) | 56.33  (46.8-65.86) | 561353.06  (516553.56-606152.57) | 14.59  (13.43-15.76) | 10632312.43  (9698659.92-11565964.94) | 276.48  (252.2-300.76) |
| 2037 | 2224290.66  (1831814.63-2616766.68) | 57.08  (47.01-67.15) | 566164.44  (519015.67-613313.21) | 14.52  (13.32-15.73) | 10759010.18  (9772505.91-11745514.45) | 276.11  (250.79-301.42) |
| 2038 | 2283728.83  (1863899.49-2703558.17) | 57.86  (47.22-68.49) | 570744.39  (521239.93-620248.86) | 14.46  (13.2-15.71) | 10883694.05  (9843256.28-11924131.82) | 275.75  (249.39-302.11) |
| 2039 | 2345019.62  (1896463.74-2793575.5) | 58.68  (47.45-69.9) | 575207.86  (523326.69-627089.03) | 14.39  (13.09-15.69) | 11008872.71  (9912960.75-12104784.67) | 275.47  (248.05-302.9) |
| 2040 | 2408776.03  (1929826.27-2887725.79) | 59.55  (47.7-71.39) | 579684.53  (525380.27-633988.8) | 14.33  (12.98-15.67) | 11136530.21  (9982972.82-12290087.6) | 275.31  (246.8-303.83) |

Abbreviations: ASR, age-standardized rate; CI, confidence interval; DALYs, disability adjusted life years.
